# Increasing Sea Surface Temperatures Driving Widespread Tropicalization in South Atlantic Pelagic Fisheries

**DOI:** 10.3390/biology14081039

**Published:** 2025-08-13

**Authors:** Rodrigo Sant’Ana, Daniel Thá, Lea-Anne Henry, Rafael Schroeder, José Angel Alvarez Perez

**Affiliations:** 1 Laboratório de Estudos Marinhos Aplicados, Escola Politécnica, Universidade do Vale do Itajaí (UNIVALI), Rua Uruguai 458, Itajaí 88302-901, Brazil; daniel.tha@kralingen.com.br (D.T.); schroederichthys@gmail.com (R.S.); angel.perez@univali.br (J.A.A.P.); 2 School of GeoSciences, University of Edinburgh, King’s Buildings, James Hutton Road, Edinburgh EH9 3FE, UK; l.henry@ed.ac.uk; 3 Centro Interdisciplinar de Investigação Marinha e Ambiental (CIIMAR), Terminal de Cruzeiros do Porto de Leixões, Avenida General Norton de Matos S/N, 4550-208 Matosinhos, Portugal

**Keywords:** climate change, tropicalization, South Atlantic Ocean, pelagic megafauna, mean temperature of the catch (MTC), marine fisheries

## Abstract

Ocean warming is leading to a tropicalization of fisheries in subtropical regions around the world. Here, we scrutinize pelagic fisheries catch data from 1978 to 2018 in the South Atlantic Ocean in search of signs of tropicalization in these highly migratory and top-of-the-food-chain fish. Through the analysis of catch composition data, thermal preferences, and climatic data, we described the temporal variability in the mean temperature of the catch and assessed the role of sea surface temperature and the Brazil Current’s transport volumes as drivers of such variability. We observed a significant increase in the mean temperature of the catches, indicating a transition towards a predominance of warm-water species, especially pronounced on the western side of the South Atlantic Ocean. This shift was further corroborated by a significant rise in the proportion of warm-water species over time. Additionally, this study observes a continuous increase in SST during the entire time series on both sides of the South Atlantic Ocean, with significant positive trends. The analysis of catch composition through ordination methods and estimates of beta diversity reveals a transition from an early scenario characterized by mostly cold-water species to a late scenario, dominated by a greater diversity of species with a prevalence of warm-water affinities. These findings underscore the profound impact of ocean warming on marine biodiversity, with significant implications for fisheries management and ecosystem services.

## Simple Summary

This study examined pelagic fisheries catch data (1978–2018) in the South Atlantic Ocean to assess the effects of ocean warming, particularly the “tropicalization” of fish populations. By analyzing catch composition, species’ thermal preferences, and climate data, the results evidenced: (i) a significant increase in the mean temperature of catches, indicating a shift toward warm-water species, especially in the western South Atlantic; (ii) a rising proportion of warm-water species over time, with the sharpest increase in the South West Atlantic; (iii) a continuous, significant rise across the region of sea surface temperatures (SST); and (iv) a catch composition shift from cold-water-dominated to warm-water-dominated, with greater species diversity in recent years. These findings highlight the major impact of ocean warming on marine ecosystems, with important implications for fisheries management and biodiversity conservation.

## 1. Introduction

Marine organisms are increasingly important in the diet of the expanding human population [1,2]. Yet, the quest for nutrient-rich foods that generate lower emissions than many terrestrial meats, also known as “blue foods” (mainly animal protein), continues to grow. Pressures from overfishing and other human activities, such as pollution, habitat destruction, and climate change, threaten the productivity of marine ecosystems, with climate-driven alterations causing particular concern [3,4].

In 2023, the global annual mean sea surface temperature (SST) reached the highest mark ever recorded by modern instruments [5]. Rising ocean heat content has unleashed changes in conditions spanning form the rise of the sea level and the expansion of well-stratified areas to decreases in salinity, dissolved oxygen levels, and primary productivity [6,7,8,9]. Marine species, including many fish stocks, have responded to warming waters by altering their phenology, expanding or shifting their distributions poleward towards higher latitudes and deeper waters, accompanied by changes in abundances, biomass, and production [10,11,12,13,14,15]. Collectively, these responses tend to alter regional species diversity and food web structures. Coupled with increasing temperatures, these responses may accelerate organisms’ metabolic rates, potentially impairing ecosystem functioning and the benefits they deliver to human societies, including those related to fisheries [15,16,17,18,19,20].

In the Global South, climate adaptation of blue food systems, including fisheries, will be critical because these foods play a vital role in nutrition, livelihoods, and revenue in these nations [21]. Pelagic fisheries for tunas and related species represent a substantial proportion of marine fish production [4,22]. These species are highly mobile, high-level predators of the pelagic systems, whose distribution and life history patterns are tuned with optimal thermal conditions and large and mesoscale biophysical processes that enhance biological productivity in the oceans and concentrate pelagic prey. Their high metabolic demands, extensive migratory patterns, and position at the top of food chains exert top–down control, regulate prey populations, and make them sentinels of large-scale oceanographic shifts [23,24]. In this sense, climate-induced oceanographic changes may drastically affect tuna populations, leaving detectable signs of these effects in their patterns of distribution (e.g., poleward movements), abundance, or biomass, for example [4,25].

**Figure 1:**
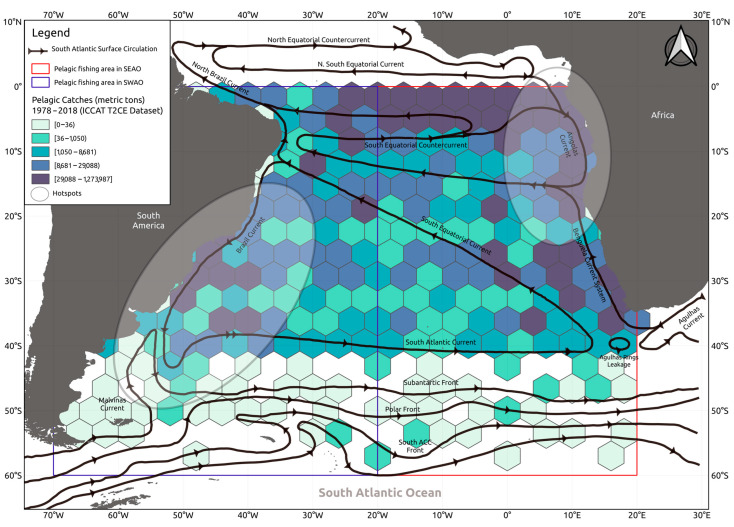
South Atlantic Ocean (SAO) and the established division between the South West Atlantic Ocean (SWAO) and the South East Atlantic Ocean (SEAO) based on general populations used by the International Commission of the Conservation of Atlantic Tuna (ICCAT). Ellipses represent ocean hotspots according to Hobday and Pecl [34]. Black arrows represented the direction of the South Atlantic Surface Circulation according to Talley et al. [42].

In the South Atlantic Ocean (SAO), the pelagic system down to approximately 1000 m water depths is under the influence of the wind-driven Subtropical Gyre [26] formed by the geostrophic flow of the Brazil Current (BC), South Atlantic Current, Benguela Current, and South Equatorial Current [27] (Figure 1). Gyre dynamics facilitate biophysical processes that concentrate biological production, aggregating pelagic species, and sustaining large-scale fishing [28,29,30,31]. In the past decades, however, global-warming-induced changes in wind regimes have altered circulation patterns of the Subtropical Gyre, modifying the spatial and temporal distribution of productive open-ocean regions with potential effects on pelagic communities. Evidence of such alterations includes (a) a southward migration of the South Equatorial Current bifurcation off NE Brazil, shifting the Subtropical Gyre system poleward [32,33,34], and (b) the poleward displacement of wind patterns leading to the southward extension of the BC and the Brazil–Malvinas Confluence [29,35,36]. In the Angola Basin and the Southwest Atlantic, between southern Brazil and northern Argentina, these circulation shifts have contributed to the formation of ocean *hotspots* (i.e., areas where surface temperatures have increased above global average in the past 50 years) [37,38], where important ecological responses have been predicted [39] and observed in small clupeid and engraulid fish and the Blackfin tuna *Thunnus atlanticus* [14,15,29]. Particularly relevant is the documentation of the ‘tropicalization’ of marine fauna in the Southwest Atlantic hotspot over the past decade, evidenced by increasing proportions of fish and shellfish species with warm-water affinities in demersal catches recorded off the coasts of southern Brazil, Uruguay, and northern Argentina [39,40,41].

Climate-driven changes in thermal conditions (e.g., hotspots) and biophysical processes around the SAO Subtropical Gyre have likely placed tuna and tuna-like populations under considerable pressure in recent decades, exacerbating shifts in their spatial distribution and abundance or biomass, also leading to alterations in species composition and ultimately the trophic structure of oceanic ecosystems [4,43,44]. Some of these large predators of the pelagic system (including sharks), for example, seem to move seasonally from feeding to spawning grounds on the highly productive Brazil–Malvinas Confluence and the tropical Atlantic (equatorial currents system), respectively [29,35,36]. These are areas known to be progressively altered in response to climate-driven changes [17], with consequences toward thermal conditions and biological productivity, all drivers of the spatial distribution of tuna and tuna-like species. Because they are highly mobile, temperature-sensitive, energy-demanding, and top–down control in pelagic food webs [45,46,47], population responses and adaptation to changing conditions may be key to understanding current and future alterations in the SAO pelagic ecosystems. These responses, in concert with intensive fishing regimes and overexploitation, impact the current and future development of oceanic fishing and ultimately the provision of nutrition, livelihoods, and revenue for countries in the Global South.

We investigate the effects of the SAO warming process in the large predatory oceanic fish, detectable through the large-scale pelagic fisheries activity on the eastern and western sides of the SAO. We analyze four-decades-long time series of pelagic catches that include multiple species with various thermal affinities. These catches include tropical tunas such as the skipjack tuna *Katsuwonus pelamis* and yellowfin tuna *Thunnus albacares* found in waters up to 30–32 °C, but they tend to prefer temperatures above 16 °C [48]. They also contain the Atlantic bluefin tuna *Thunnus thynnus* and the albacore *Thunnus alalunga*, which have affinity for cooler waters; the former feeds extensively in waters below 10 °C and can be physiologically stressed by temperatures above 28–29 °C [49,50]; the latter prefers waters between 10° and 20 °C but tends to seek waters with temperatures above 24 °C for reproduction while maintaining their vertical migration during reproductive events for thermoregulation [51,52]. We considered these thermal preferences to calculate the mean temperatures of the catch [40] and analyzed its temporal variability, searching for patterns modulated by increasing sea surface temperatures, consistent with the ‘tropicalization’ process of the SAO [41].

## 2. Materials and Methods

### 2.1. Catch Data, Thermal Preferences, and Climatic Data

Historical records of pelagic fishing catch data were obtained from the International Commission for the Conservation of the Atlantic Tuna [ICCAT database CATDIS and T2CE available at https://iccat.int/en/ (accessed on 1 April 2022)]. While the archive spans back to the 1950s, this study focused solely on data collected from 1978 through 2018. The study area was geographically bounded between latitudes 0° and 60° S and laterally divided at 20° W to distinguish the South West (SWAO) and South East Atlantic Ocean (SEAO) sectors (see Figure 1). Recorded fishing operations involved the use of longlines, purse seines, bait boats, and handlines and were conducted by fishing fleets of distinct nations. Annual reported catches varied, ranging from approximately 219,511 metric tons in 1979 to nearly 411,185 tons by 2018. Landings were dominated by tuna species (e.g., *Thunnus thynnus*, *Thunnus alalunga*, *Thunnus obesus*, *Thunnus albacares*, *Katsuwonus pelamis*, *Auxis thazard*, *Thunnus atlanticus*, *Auxis rochei*, *Euthynnus alletteratus*), billfish (e.g., *Makaira nigricans*, *Istiophorus albicans*, *Tetrapturus pfluegeri*, *Xiphias gladius*), and large pelagic sharks (e.g., *Prionace glauca*, *Lamna nasus*, *Isurus oxyrinchus*).

Annual fishing data contained information from 35 catch categories, defined either by single species or groups of species (e.g., ‘*Thunnus* spp.’). For this study, only single-species categories were considered (29 species), which jointly represented more than 95% of the total reported biomass. *Thermal affinities* for each considered species were assigned based on global datasets compilations derived from the species distribution ranges and sea surface temperature maps made available by Cheung et al. [40] and in FishBase [53]. An overall mean thermal preference of 24.03 °C was calculated across all species and used as a reference threshold to distinguish between taxa associated with warm waters (16 species with thermal preferences above the overall mean) and those favoring colder environments (13 species with thermal affinities below this overall mean). The “Mean Temperature of the Catch (MTC)” was subsequently calculated for each year (*y*) following the methodology proposed by Cheung et al. [40], incorporating both catch data and species-specific temperature preferences:

$${MTC}_{y}=\frac{\sum_{i}^{n}T_{i}C_{i,y}}{\sum_{i}^{n}C_{i,y}}$$

where *n* is the total number of species recorded in one year, *T_i_* is the temperature preference (in °C) of the *i*th species, and *C_i,y_* is the recorded catch (in tons) of the *i*th species in the *y*th year. While using catch tonnage may potentially bias species contributions to the MTC calculation due to interspecific differences in body mass, the biomass-based approach is fit for purpose since (*i*) it is consistent with the original MTC methodology proposed by Cheung et al. [40]; (*ii*) it reflects the actual fishing pressure and economic importance of each species in the fishery, and (*iii*) it captures the real-world impact species shifts on fishery yields and ecosystem services, as discussed in several studies around the world [40,41,54,55,56,57,58,59,60].

Sea Surface Temperature (SST) was used as an explanatory variable for MTC trends in both the South East (SEAO) and South West Atlantic Ocean (SWAO). SST data were sourced from the INALT20 model [61], covering the period from 1978 to 2018, and mean temperatures were computed across 0.25° × 0.25° grid cells in the upper 100 m of the water column throughout the SAO. In addition, for the SWAO, interannual variations in the MTC were also compared against the annual transport volume of the Brazil Current (BCt) near the Brazil–Malvinas confluence. These transport estimates (in Sverdrup’s) were available for 2000–2017 and derived from the high-resolution (1/12°) global Mercator Ocean reanalysis (GLORYS12) from Copernicus Marine Environment Monitoring Service [CMEMS, http://marine.copernicus.eu/ (accessed on 1 April 2022)], as reported by Artana et al. [36]. To ensure comparability, all annual values were normalized relative to their respective long-term means and subsequently expressed as anomalies.

Temporal dynamics of MTC, SST, and BCt were assessed by applying linear and non-linear regression models across the time series. To implement the nonlinear models, a Generalized Additive Model (GAM) was used, in which the response variables were modeled as a function of time using a smoothing function represented by a penalized cubic spline with a maximum number of five bases (k = 5). This specification defines the functional space in which the smooth function will be estimated, allowing the complexity of the fitted curve to be determined empirically by the data (e.g., data-driven). The penalty associated with smoothing controls the degree of effective flexibility of the fitted function, preventing overfitting by restricting the number of degrees of freedom used. Thus, the model can fit from an approximately linear function to a more complex nonlinear curve, as justified by the data structure [62,63]. The choice of model structure (e.g., linear or nonlinear) was evaluated by comparing the adjusted R-squared and the Akaike and Bayesian information criteria. Residual analysis was also observed between the two structures. The final decision regarding the structure also considered the purpose of the investigation, as proposed by Tredennick et al. [64]. Thus, even though there was a slight difference in the adjusted R-squared and AIC values favoring the nonlinear structure for the SST response variable modeled on the eastern side of the South Atlantic Ocean (SEAO), the Bayesian information criterion and residual analysis did not show the same behavior. For the other response variables applied on both sides of the South Atlantic Ocean, it was not possible to observe gains in the application of non-linear models such as those evaluated here. Therefore, it was decided to maintain the structure defined by the linear regression models and, in turn, observe the general trends of the time series over the observed period. This choice also permitted greater comparability of these effects with other studies conducted worldwide [40,41,54,55,56,57,58,59,60] (for more details, see Supplementary Material Table S1 and Figures S11–S15).

After the definition of model structure as linear regression models, we evaluated the sensitivity of the estimated MTC trends to individual species by systematically removing each species from the dataset and reassessing the trend. This approach aimed to determine whether variability in the catch of a single, abundant species (e.g., especially those heavily targeted in certain years) could disproportionately influence the overall MTC signal. Such an effect could potentially obscure broader trends derived from the full multispecies assemblage [39]. The approach involved iteratively recalculating MTC regression models while excluding one species at a time. For each scenario, the estimated slope of the regression was compared to that derived from the model fitted with the full dataset to evaluate the robustness of the temporal trend. In parallel, the influence of environmental drivers (SST and BCt) on MTC variability was tested using time-lagged linear models (lags ranging from 0 to 4 years), using the R package ‘dynlm’ version 0.3.6 as proposed by Zeileis [65] and discussed by Pfaff [66]. This method allows for the incorporation of possible delayed ecological responses in the species composition in response to environmental changes. The performance and selection of the time-lagged linear models were evaluated based on three different criteria: (*i*) the minimum Akaike Information Criterion (AIC) value, (*ii*) the highest R-squared value, and (*iii*) the statistical significance of the estimated slope parameter. To explore spatial heterogeneity in responses, temporal trends of MTC were also compared between the SWAO and SEAO using an Analysis of Covariance (ANCOVA), with particular focus on differences in model slopes.

### 2.2. Catch Composition Analysis

Changes in species composition across the 41-year time series were analyzed separately for the SWAO and SEAO using ordination methods and beta diversity metrics. A Multiple Regression Tree (MRT) analysis was applied to Hellinger-transformed annual species catches (biomass data) implemented via the ‘mvpart’ R package [67]. The optimal tree size, in terms of the number of splits, was selected based on cross-validation error, balancing model fit with parsimony [35]. To visualize temporal patterns of compositional change, a Principal Coordinate Analysis (PCoA) was performed on the resulting Hellinger distance matrix, projecting individual years into a two-dimensional Euclidean space. This process allowed for the exploration of patterns of similarity/dissimilarity among years and among groups of years as previously defined by the MRT analysis [68].

The overall non-directional beta diversity (BDtotal) was calculated by computing the total sum of squares (SStotal) of the years vs. species matrix and the total variance by dividing SStotal by *n* − 1. This metric was subsequently decomposed into the relative contributions of individual years (YCBD) and species (SCBD), following the framework proposed by Legendre and De Cáceres [69]. The significance of YCBD values was assessed via 999 random permutations of the matrix columns using the beta.div function in the ‘adespatial’ R package version 0.3.23 [70], allowing for detection of years with marked shifts in species composition and the taxa most responsible for these changes. Temporal shifts in catch composition were assessed using Temporal Beta Diversity indices (TBI), calculated via the TBI function from the ‘adespatial’ R package [70]. Pairwise dissimilarities between years were quantified using percentage difference indices, which were then partitioned into components representing species gains (1 > TBI > 0) and losses (0 > TBI > −1) [71]. The computed difference between gains and losses was evaluated using a paired *t*-test. To explore the temporal structure, patterns of gains and losses between periods (e.g., similar groups of years previously identified via ordination methods) were investigated by analyzing catch variability of individual species and thermal preferences. All analyses were conducted using the language and environment for statistical computing, R 4.4.2 [72].

## 3. Results

### 3.1. Catch Composition and Thermal Preferences

Catches reported by pelagic fisheries operating in the SAO varied from 219,511 t in 1979 to 411,185 t in 2018. In the SEAO, catch volumes increased continuously along the reported period, being significantly larger than those reported in the SWAO, consistent with patterns of increased biological productivity derived from the continuous upwelling systems established along the South Atlantic African coast [73,74]. Actinopterygii and elasmobranchs accounted for 89.7% and 10.3% of the reported biomass in the study period, respectively. The reported catches were overwhelmingly dominated by skipjack, yellowfin, bigeye, and albacore tunas, which collectively contributed more than 90% of the total biomass recorded. The blue shark (*Prionace glauca*) alone constituted 88% of all elasmobranch catches (Figures S1 and S2).

**Figure 2:**
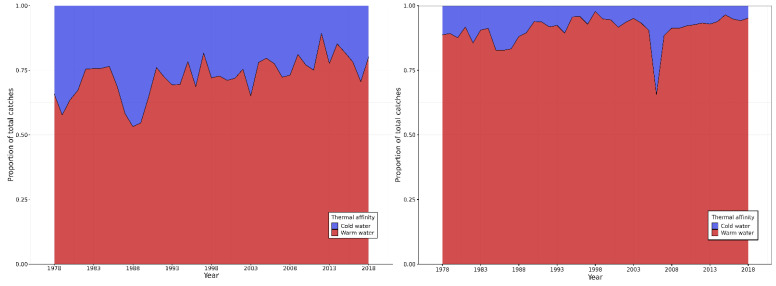
Annual variability in proportion of species with cold- and warm-water affinities in the catches of the pelagic fisheries in SAO monitored ICCAT between 1978 and 2018. SWAO on the left and SEAO in the right. Colors represent “warm-” (thermal preferences > 24.03 °C) and “cold-” (thermal preferences < 24.03 °C) water affinities.

Warm-water species, defined as those with a thermal preference above 24.03 °C, included 57.7% of the Actinopterygii species and one of the three elasmobranch species reported. The overall catch composition consistently showed a 7.3:1 ratio of species with warm- and cold-water affinities throughout the time series (SWAO 2.8:1; SEAO 10.2:1). However, there was a significant increase in the proportion of warm-water species over time across the entire SAO (a = 0.0016; b = −2.3125; *p*-value = 0.0113; r^2^ = 0.1536). This increase was particularly pronounced in the SWAO, where the relative occurrence of warm-water species rose sharply (a = 0.004; b = −7.1165; *p*-value = 0.00004; r^2^ = 0.3563). No such trend was observed in the SEAO (a = 0.0013; b = −1.7828; *p*-value = 0.0631; r^2^ = 0.0858) (Figure 2).

### 3.2. Mean Temperature of the Catches

**Figure 3:**
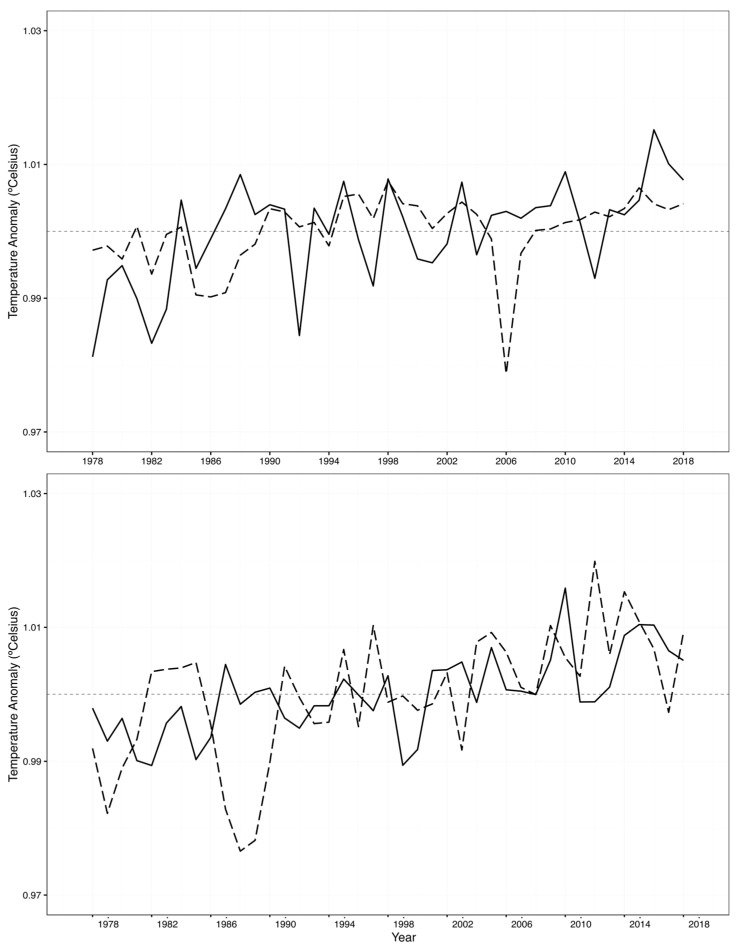
Annual variability in the MTC (dashed line) of the pelagic fisheries in the SAO monitored by ICCAT. SSTs (solid line) are superimposed. **Top**—SWAO; **bottom**—SEAO.

A consistent increase in annual MTC was observed across the 41-year series, with statistically significant trends detected on both sides of the South Atlantic Ocean (Table 1, Figure 3). Although both sides showed significant signs of increase, the patterns were statistically distinct on the two sides of the South Atlantic Ocean (ANCOVA: *p*-value = 0.0135; for model check assumptions, see Figures S3–S5). In the SWAO, MTC ranged from 24.59 °C to 25.68 °C and exhibited a significant increasing trend at a rate of 0.012 °C per year (*p* = 0.00004; see Table 1 and Table S2; for model check assumptions, see Figures S6–S8). A similar, though more moderate, trend was detected in the SEAO, where the MTC varied between 25.19 °C and 25.93 °C, increasing at a rate of 0.0042 °C per year (*p* = 0.02; Table 1 and Table S2; for model check assumptions see Figures S9 and S10).

The upward trends in the MTC observed throughout the study period remained robust on both sides of the SAO, even when individual species were systematically excluded from the linear model, one at a time (Table 2). An exception occurred in the SWAO: removing albacore led to a significant negative trend.

Sea surface temperature exhibited a consistent increasing trend across the entire time series on both the western and eastern sectors of the SAO. At the SWAO, the SST fluctuated between 23.4 and 24.1 °C and showed a significant warming trend of 0.008 °C per year (Table 1). A similar pattern was observed in the SEAO, where SST ranged from 22.2 to 23.0 °C, also increasing at a comparable rate of 0.008 °C per year over the study period (Table 1). In the SWAO, all models indicated a positive relationship between SST and MTC time-series. However, only those incorporating 2- and 4-year time-lags were statistically significant (Table 3, *p*-value = 0.03 and *p*-value = 0.003, respectively). The model with a 4-year lag yielded the highest r-squared value, suggesting it offers a better fit and may reflect a delayed community-level response to environmental warming. Similarly, it exerted a positive influence on MTC trends in the SWAO, being statistically significant when no time-lags were added (*p*-value = 0.005). In the SEAO, SST also showed a significant positive effect on MTC, but only in the model incorporating a 4-year lag (*p* = 0.019; Table 3).

### 3.3. Catch Composition Analysis

**Figure 4:**
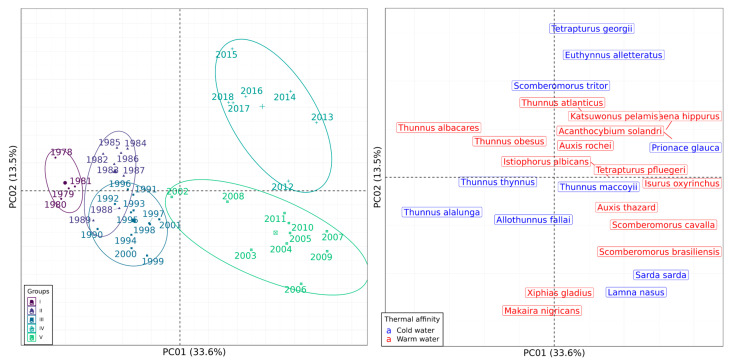
Analysis of the catch composition of the pelagic fisheries in the SWAO monitored by ICCAT between 1978 and 2018. Principal Coordinate Analysis ordination diagram representing the spatial distribution of years included in the time-series (**left**) and of the species present in the catch (**right**) according to scores of the first two extracted axes. Encircled years (colors and symbols) represent groups discriminated by the Multiple Regression Tree procedure.

**Figure 5:**
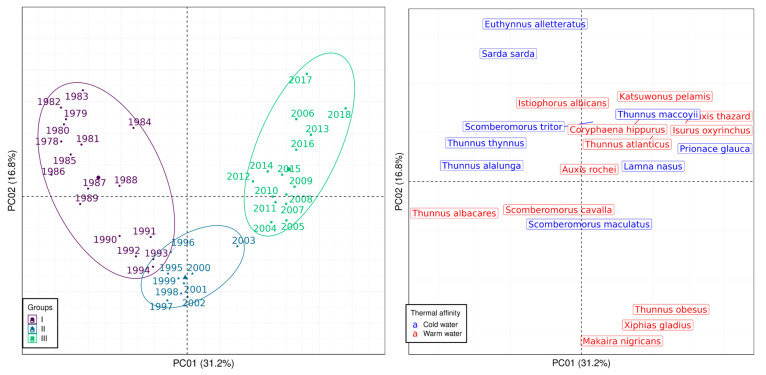
Analysis of the catch composition of the pelagic fisheries in the SEAO monitored by ICCAT between 1978 and 2018. Principal Coordinate Analysis ordination diagram representing the spatial distribution of years included in the time-series (**left**) and of the species present in the catch (**right**) according to scores of the first two extracted axes. Encircled years (colors and symbols) represent groups discriminated by the Multiple Regression Tree procedure.

The first two axes of the PCoA jointly accounted for 48% of the total variance in catches in the SWAO and 47.1% in the SEAO. Based on the temporal structure, the 41 years were clustered into five groups in the SWAO and three in the SEAO (Figure 4 and Figure 5, respectively; see Table S3 and Figures S18 and S19).

In both regions, the identified groups reflected a temporal shift from an earlier period dominated by fewer, primarily cold-water species (clusters located on the left hemiplane), to a later period marked by greater species diversity and a predominance of warm-water affinities in the catches (clusters on the right hemiplane). In the SWAO, Groups I, II, and III encompassed the first 24 years of the time-series and were characterized by the high catch biomass of cold-water species such as *Thunnus alalunga*, *Thunnus thynnus*, and *Allothunnus fallai*, along with warm-water species like *Thunnus obesus* and *Thunnus albacares* (Figure 4). In contrast, Groups IV and V corresponded to the more recent years (2002–2018) and were defined by a marked increase in the biomass of warm-water species in the catches (Figure 4).

In the SEAO, Groups I (1978–1994) and III (2004–2018) comprised years with markedly contrasting species compositions, while Group II represented a transitional period between them. Group I was characterized by catches dominated by four cold-water species (*Euthynnus alletteratus*, *Sarda sarda*, *Thunnus thynnus*, and *Thunnus alalunga*), along with two warm-water species (*Thunnus albacares* and *Scomberomorus cavalla*). In contrast, Group III encompassed 15 species, the majority (11) of which exhibited warm-water affinities, including *Katsuwonus pelamis*, *Istiophorus albicans*, *Coryphaena hippurus*, *Tetrapturus pfluegeri*, *Auxis thazard*, *Thunnus atlanticus*, *Acanthocybium solandri*, *Isurus oxyrinchus*, *Thunnus obesus*, *Xiphias gladius*, and *Makaira nigricans*. The remaining four species in this group (e.g., *Thunnus maccoyii*, *Scomberomorus tritor*, *Prionace glauca*, and *Lamna nasus*) were cold-water affiliated (Figure 5).

Total non-directional beta-diversity indices were estimated across the entire 41-year period for both sectors of the South Atlantic Ocean (SAO). Overall, the beta-diversity indices revealed subtle interannual variation in species biomass on both sides of the SAO (SWAO = 0.0928; SEAO = 0.0371). On the other hand, regional differences in total beta-diversity indices indicated that variability in species biomass over time is greater in the SWAO (2.5 times) than in the SEAO. The decomposition of total beta diversity into year-specific contributions (YCBD) revealed no statistically significant patterns in either region (Figure S16). Except for 1978, 1979, and 1980 in the SWAO, and 2006 in the SEAO, when YCBD values were relatively higher, year-to-year contributions to beta diversity remained relatively constant throughout the time series. When partitioned by species contributions (SCBD), three species accounted for over 65% of the total beta diversity in the SWAO: *Katsuwonus pelamis* (warm-water affinity), *Prionace glauca*, and *Thunnus alalunga* (both cold-water affinity). In the SEAO, five species collectively explained more than 65% of the total beta diversity, including the cold-water species *Prionace glauca*, *Scomberomorus tritor*, and *Thunnus alalunga*, and the warm-water species *Thunnus obesus* and *Thunnus albacares*.

**Figure 6:**
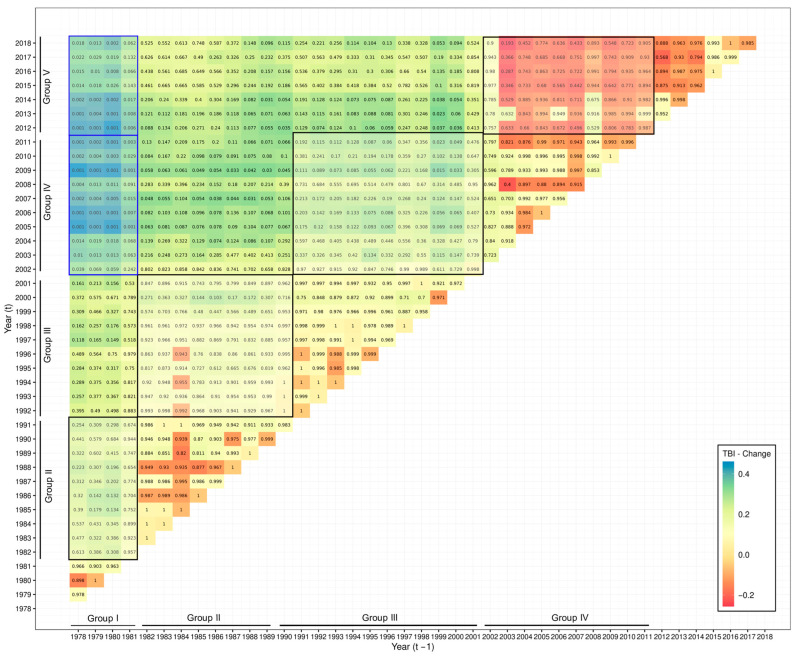
Analysis of the catch composition of the pelagic fisheries in the SWAO monitored by ICCAT between 1978 and 2018. TBI was computed between all possible pairs of years within the time series considered (Biomass gains—TBI > 0, Biomass losses Biomass gains—TBI < 0). Boxes enclose comparisons between years included in three groups discriminated by the multiple regression trees and principal coordinate analysis.

**Figure 7:**
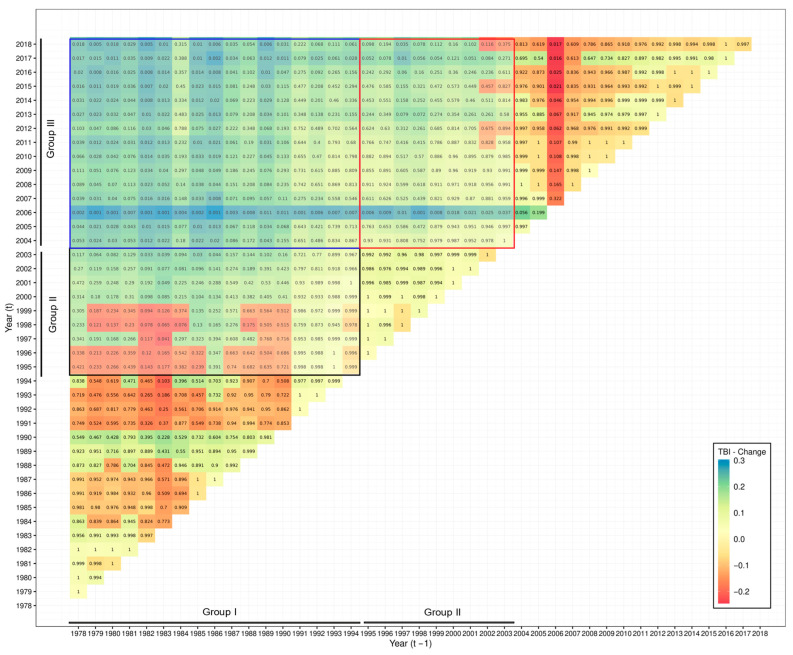
Analysis of the catch composition of the pelagic fisheries in the SEAO monitored by ICCAT between 1978 and 2018. TBI computed between all possible pairs of years within the time series considered (Biomass gains—TBI > 0, Biomass losses Biomass gains—TBI < 0). Boxes enclose comparisons between years included in three groups discriminated by the multiple regression trees and principal coordinate analysis.

TBIs were also computed for all possible pairs of years within the time series for both sides of SAO (Figure 6 and Figure 7). The resulting values were interpreted according to the distribution of positive (species biomass gains) and negative (species biomass losses) values, comparing the groups of years defined by the PCoA (Figure 4 and Figure 5). In the SWAO, pairwise comparisons of years within Group I against Groups IV and V exhibited the most significant changes, indicating only biomass gains of species in the catches (Figure 6). This pattern was largely driven by the appearance or increasing biomass of warm-water species in the catches, as highlighted by the PCoA results. In the SEAO, a gradual shift was observed over time, from predominantly losses to predominantly gains in species biomass (Figure 7). The most pronounced and statistically significant changes occurred between Groups I and III, with recent years (Group III) exhibiting substantial gains in species biomass (Figure 7). The results of TBI also corroborate the pattern observed in PCoA, with an increase in biomass or even the entry of new species with warm-water affinity in the catches.

## 4. Discussion

The analysis of pelagic fisheries data from the SAO between 1978 and 2018 reveals a rising trend in MTC, accentuated in the last two decades, across both the SWAO (24.59–25.68 °C) and the SEAO (25.19–25.93 °C). This indicates that both western and eastern sides of the SAO contain signs of tropicalization of pelagic assemblages: biomass losses of mostly cold-water species and/or biomass gains of mostly warm-water species. These changes in the composition of catches may also be linked to changes and movements of the prey of these large predators, which, due to their migratory and adaptive capacity to thermal changes, may be responding to changes in food availability before responding to direct changes in environmental temperature. However, both possibilities are likely and result from changes in the environment conditioned by climate change that reflect their impacts on all ecosystems.

On the other hand, because these trends derive from commercial catches, it is critical to consider a potential artefact introduced by economic processes (e.g., market demands) driving fishing fleet dynamics and consequently affecting variability in catch composition. Essentially, such an artefact would most likely produce a false tropicalization effect in the MTC temporal variability if fishing fleets shifted their spatial strategy to increase catches of market-demanded species with an affinity for warm waters, such as tropical tunas (e.g., Skipjack tuna, Yellowfin tuna, Bigeye tuna). Thus, these changes can be driven by changes in fishing strategies (top–down: anthropogenic pressure via fishing activity); however, as described by Liu et al. [32], changes based on environmental and climatic forcing’s should not be disregarded (bottom–up), demonstrating that changes in species composition are more sensitive to climatic and environmental changes in the ecosystem, especially in the SAO. Changes in the environment, derived from climatic influences, are preponderant factors for changes in species composition in catches and, in general, can have a more rapid effect on the behavior of the activity than market pressure as a whole [75,76,77,78]. Nonetheless, it was demonstrated that the increasing trends in MTC remained significantly positive in the adjusted models even when these species were iteratively removed from the analysis, one by one. The results of this sensitivity analysis implied that potential shifts in market demands did not affect the MTC series significantly, supporting the robustness of tropicalization signs. On the other hand, it is important to note that adjusted models were sensitive to one species, the albacore (*Thunnus alalunga*), which, when removed from both SWAO and SEAO MTC series, produced a significant negative trend (Table 3). Important catches of this “cold water tuna” cooled down the MTC during the early years of the time series, but the species became progressively scarcer in the catches thereafter, significantly accounting for MTC positive trends, yet there is no evidence that this was the outcome of any market direction [32]. Additionally, this effect may also be associated with the ability of albacore to perform vertical movements as a form of thermoregulation, given the species’ greater intolerance to warm waters, thus descending to deeper and colder layers of the water column, no longer being directly available for fishing activity [53,61].

Discriminating the effects of human-related drivers of fishing activity from ecosystem-related ones is particularly complex in a multinational fishery spread throughout decades over an entire ocean basin. In the SWAO, climate-induced changes in the marine environment since the 1950s have influenced fishery regime shifts, despite ecosystem effects induced by fishing (e.g., overfishing, top–down control) [32]. Environmental variability, particularly ocean warming, has exerted global effects on the spatial distribution, migratory phenology, and population dynamics of highly migratory pelagic species such as tunas, swordfish, billfishes, and sharks [79,80,81,82]. Rising sea temperatures elevate metabolic rates in ectothermic organisms, accelerating growth until thermal tolerance thresholds, beyond which physiological fitness declines and mortality risks escalate [45,46,83]. While large pelagic predators exhibit some acclimation capacity to thermal variability, their abundance and distribution remain intrinsically linked to species-specific environmental niches, habitat characteristics, and spatial overlaps with fishing activities [47]. Consequently, environmental fluctuations directly influence the catchability of these species, compelling pelagic fishing fleets to adapt their operational strategies accordingly [76,77,78].

When faced with such environmental changes, adaptation of fishing fleets is usually autonomous and involves technology, targeting new species and/or exploration of different fishing grounds, contingent on market dynamics, regulatory path dependencies and even personal preferences [84,85,86]. Since industrial capture fisheries, especially long-established fleets with large capital invested and consolidated upstream integration, are primarily supply-driven [87], market dynamics are secondary drivers of change. In the Bay of Biscay, for example, short-term tactics of the pelagic fisheries were not shown to be influenced by market prices, and even long-term decisions prioritized métier attractiveness over purely economic considerations [84]. Empirical evidence from the Western Mediterranean, South Atlantic, Indian, and Eastern Pacific Oceans also demonstrates that strategic fishing shifts are predominantly reactive to ecological pressures rather than proactive market-driven initiatives. Increases in sea temperature, salinity shifts, and declining primary productivity have spurred changes in fleet dynamics, as observed in the Mediterranean small pelagic fisheries and Atlantic bluefin tuna fisheries [88,89].

In this study, the observed signs of tropicalization of pelagic fauna were particularly evident in the contrasting patterns of early against late years of the time series. In general, on both sides of SAO, catches in the early years included abundant species with cold-water affinity, a pattern that changed gradually up to the most recent years, where an increase in the biomass of warm-water species in the catch composition was salient. In the SWAO, these gains were mostly defined by the occurrence/biomass of warm-water species (*Katsuwonus pelamis*, *Scomberomorus brasiliensis*, *Xiphias gladius*, *Thunnus obesus*, *Thunnus albacares*, and *Scomberomorus cavalla*) and by the main reduction in cold-water species like *Thunnus alalunga* (Figure S17). This reduction in the participation of *Thunnus alalunga* is not directly linked to reductions in the stock’s biomass, as its catches have remained below the maximum sustainable yields estimated for the stock in the SAO [90]. However, the species is known for its ability to perform vertical movements as a form of thermoregulation, given its greater intolerance to the influence of warm waters, and, therefore, is no longer available for catching by surface gear, even though there have been no significant changes in its abundance [48,49]. In the SEAO, the species changes were defined by the gains of warm-water species like *Thunnus obesus*, *Thunnus albacares*, *Katsuwonus pelamis*, *Xiphias gladius*, and *Auxis thazard*, and by the losses of cold-water species such as *Thunnus alalunga* and *Thunnus thynnus* (Figure S17). Monllor-Hurtado et al. found a strong increasing trend in the relative participation of the *Katsuwonus pelamis* and *Thunnus albacares* in total pelagic catches in the SAO, even when the total catches had descended in the recent period [4]. These findings indicated that an increase in the relative participation of tropical tunas in pelagic catches has been more evident in subtropical regions of the Atlantic Ocean, supporting the effects of the tropicalization observed in the present study.

The pelagic catch composition tended to change more rapidly in response to ocean warming in the SWAO than in the SEAO; the MTC increase in the former (0.12 °C per decade) was threefold higher than in the latter (0.04 °C per decade). In both regions, MTC was significantly explained by a similar SST increase in the period (~0.008°.yr^−1^), but such a relationship was weaker in the SEAO (*p* = 0.018) and stronger in the SWAO (*p* = 0.003). Both fishing areas have been affected by climate-change-induced alterations in the South Atlantic Subtropical Gyre. Current knowledge of these processes, however, is not fully comprehended, hampering regional comparisons. Recent studies have concluded that the SWAO hotspot is a result of a southward expansion of the warm waters of the Brazil Current induced by a poleward displacement of wind patterns of the South Atlantic [35,91]. Artana et al. [36] demonstrated that annual volume transports of the Brazil Current (BCt) increased from 1998 to 2016, displacing the Brazil–Malvinas Confluence southwards (0.6–0.9° latitude per decade), producing a general increase in the sea surface temperatures in the region. These findings correspond with the intensified SST anomalies and MTC increase in the SWAO, particularly noting a 2-year lag in the response to BCt. Gianelli et al. reported increasing mean temperatures of demersal catches in the Argentinian Uruguayan Common Fishing Zone (AUCFZ, ~34°–40°S) between 1973 and 2017. Considering that this is a subtropical–temperate transition zone, these authors suggested that, while species were naturally adapted to environmental fluctuations, accelerated warming could lead to significant shifts in ecological community composition and structure, supporting the temperature anomalies found in recent decades [37,38,39]. In addition, Rodrigues et al. [92] found that marine heat waves (MHW) have become more frequent and persistent in the SWAO in the last two decades, increasing from up to 6 days longer per decade to frequency values varying between 10 and 30 days per year per decade.

In the SEAO, alterations in the dynamics of the Angola–Benguela upwelling system have been attributed to global climate change [37,93]. However, its numerous upwelling cells, such as the Cape Point cell, Cape Columbine cell, Namaqua cell, Luderitz cell, Central Namibian cell, Northern Namibian cell, and Cunene cell [94], may mitigate warming effects when compared to the SWAO. On the other hand, in the subregion known as the southeastern tropical Atlantic region (SETA—10–20° S, 5° W–15° E), which has subtropical characteristics and encloses a SEAO hotspot, the weakening of alongshore southerly winds has caused a reduction in coastal upwelling, the southward expansion of stratified tropical waters, and the southward shift of the Angola–Benguela front [31]. Along the SEAO, marine heatwaves have become more frequent everywhere by up to 15 days per year per decade [92]. The extent of these oceanographic changes in the SEAO is analogous to that in the SWAO; however, these effects were less intense in SEAO [92]. Their impact on megafauna assemblages follows the same tendencies observed in both ecosystems in this study.

Our findings underscore the impact of climate change on marine ecosystems, aligning the SAO’s pelagic megafauna data with other observations of marine species’ redistribution in response to a warming ocean across different ecosystem compartments, oceanic layers, and specific regions [4,39,40,41,56,95,96,97]. Specifically, Monllor-Hurtado et al. [4] observed an uptrend in the proportion of tropical tunas (species preferring warmer waters) relative to total catches, particularly in the SAO’s subtropical zones. They proposed that this trend was exacerbated by the poleward migration of tropical tuna populations in response to ocean warming. The rates of MTC increase found on the SAO, however, are lower than those estimated globally (0.19 °C per decade) and for non-tropical regions (0.23 °C per decade) [40], as well as in various global ocean regions [40,56,94]. This discrepancy in rates may partly result from this study’s focus on large migratory pelagic species, whose dynamic movements could potentially dampen observed MTC trends, which may also explain the considerable higher rates reported for the less mobile demersal megafauna (0.41 °C yr^−1^) in the SWAO [41].

## 5. Conclusions

Despite the challenges posed by using historical catch data from pelagic fisheries, we have shown it to be an indicator of the global climate’s impact on the SAO pelagic ecosystem. The changes in catch compositions, and consequently in the structures of the studied communities, appear to be associated with shifts in oceanographic circulation patterns observed in both sides of the South Atlantic Ocean. The high frequency of upwelling zones on the eastern side of the Atlantic seems to have a buffering effect on the response of species when compared with the western side of the South Atlantic Ocean. Overall, the evidence presented here highlights the potential effects already observable of ocean warming and its impacts on the provisioning ecosystem service, specifically fishing, in the South Atlantic Ocean. The data not only reflect current changes but also hint at potential future shifts in the landscape of fisheries, including their economic consequences. Should the tropicalization trend towards a higher proportion of warm-water species continue into the coming decades, what will the response of fishers be to the next pelagic assemblages on both the western and eastern sides of the SAO? Since fisheries are a key ecosystem service, what are the economic and nutritional consequences of a disruption in the ocean’s capacity to provide it? The increase in warm-water species may also affect the structure and function of the pelagic ecosystem, potentially altering food web dynamics, nutrient cycling, and fishery yields. Addressing these questions will necessitate further comprehensive analyses incorporating spatial data and economic factors related to fisheries.

## Figures and Tables

**Table 1:** Analysis of temporal trends in the variability in the MTC of the pelagic fisheries in SAO between 1978 and 2018, SST and BCt). The ranges and slopes of fitted linear models are indicated (MTC yr^−1^, SST yr^−1^, BCt yr^−1^) for the entire time series (1978–2018).

Ocean Side	MTC Range (°C)	MTC/yr (°C)	*p*-Value	r^2^	Adj. r^2^
SWAO	24.59–25.68	0.0120	0.00004	0.3586	0.3422
SEAO	25.19–25.93	0.0042	0.0201	0.1309	0.1087
**Ocean Side**	**SST Range (°C)**	**SST/yr (°C)**	***p*** **-Value**	**r^2^**	**Adj. r^2^**
SWAO	23.38–24.01	0.0079	0.000002	0.4477	0.4336
SEAO	22.19–22.96	0.0080	0.0001	0.3186	0.3012
**Ocean Side**	**BCt Range (Sv)**	**BCt/yr (Sv)**	***p*** **-Value**	**r^2^**	**Adj. r^2^**
SWAO	−30.10–−14.19	0.366	0.0003	0.450	0.4255
SEAO	-	-	-	-	-

**Table 2:** Sensitivity analysis of temporal trends estimated for MTC of the pelagic fisheries on both sides of SAO monitored between 1978 and 2018. The species listed below are those that, when excluded from the analysis, changed the slope of the fitted linear model by more than 5% (positive or negative). In bold are slopes and *p*-values estimated with all species included. Thermal affinities are included: warm > 24.03 °C; cold < 24.03 °C.

SAO Side	Species	Thermal Affinity	Slope	*p*-Value	Slope Change (%)
**SWAO**			**0.0120**	**0.000035**	
	*Prionace glauca*	Cold	0.0209	0.000000002	74.378
	*Thunnus albacares*	Warm	0.0171	0.000001	43.192
	*Thunnus obesus*	Warm	0.0167	0.000003	39.583
	*Scomberomorus brasiliensis*	Warm	0.0109	0.000141	−8.929
	*Katsuwonus pelamis*	Warm	0.0090	0.007880	−25.070
	*Thunnus alalunga*	Cold	−0.0115	0.00000000037	−196.328
**SEAO**			**0.0042**	**0.0201**	
	*Thunnus albacares*	Warm	0.0097	0.0003	132.573
	*Prionace glauca*	Cold	0.0067	0.0001	60.373
	*Katsuwonus pelamis*	Warm	0.0051	0.0455	21.649
	*Scomberomorus tritor*	Cold	0.0046	0.0010	10.319
	*Thunnus maccoyii*	Cold	0.0045	0.0123	8.786
	*Isurus oxyrinchus*	Warm	0.0043	0.0171	2.648
	*Thunnus obesus*	Warm	0.0039	0.0406	−5.952
	*Auxis thazard*	Warm	0.0038	0.0353	−9.865
	*Euthynnus alletteratus*	Cold	0.0034	0.0524	−17.728
	*Thunnus alalunga*	Cold	−0.0024	0.0856	−158.109

**Table 3:** Results for linear models fitted between the MTC (response variable) and the explanatory variables: SST and BCt with and without time lags (years). R^2^ and Akaike Information Criteria values computed for each model are informed.

SAO Side	Variable	Time Lag (yr)	Slope	SE	*p*-Value	r^2^	AIC
**SWAO**	SST	0	0.4570	0.2603	0.0869	0.0733	1.04
		1	0.3588	0.2676	0.1879	0.0452	2.59
		2	0.5761	0.2513	0.0277	0.1244	−3.54
		3	0.5283	0.2640	0.0530	0.1001	−2.95
		4	0.8378	0.2581	0.0026	0.2314	−8.30
	BCt	0	0.02325	0.00751	0.00511	0.2940	−20.69
		1	0.01101	0.00847	0.20619	0.0685	−14.70
		2	0.00749	0.00851	0.38849	0.0340	−13.69
		3	0.01670	0.00850	0.06281	0.1553	−15.09
		4	0.00961	0.00933	0.31505	0.0504	−12.69
**SEAO**	SST	0	0.1970	0.1256	0.1249	0.0593	−43.56
		1	0.1138	0.1312	0.3913	0.0194	−39.99
		2	0.2461	0.1312	0.0686	0.0868	−40.81
		3	0.0966	0.1462	0.5132	0.0120	−36.26
		4	0.3428	0.1388	0.0186	0.1483	−39.66

## Data Availability

The datasets analyzed during the current study are available in the (a) International Commission for the Conservation of the Atlantic Tunas repository [ICCAT database CATDIS and T2CE available at https://iccat.int/en/ (accessed on 1 April 2022)] and (b) github repository developed to maintain the species thermal affinities dataset and consolidated environmental timeseries (available at https://github.com/rodrigosantana/PelagicTropicalizationSAO (accessed on 1 April 2022)). Additional details regarding datasets used in the current study are available from the corresponding author upon request.

## Supplementary Materials

The following supporting information can be downloaded at: https://www.mdpi.com/article/10.3390/biology14081039/s1, Table S1: Summary of the comparison between Linear and Non-Linear models applied to the Mean Temperature of the Catch (MTC), Sea Surface Temperature (SST) and Annual Transport Volume of the Brazil Current (BCt) time-series. South East Atlantic Ocean (SEAO)—MTC and SST were evaluated; South West Atlantic Ocean (SWAO)—MTC, SST and BCt were evaluated. For each method were presented the adjusted r-squared (Adj. r^2^), Akaike Information Criteria (AIC) and Bayesian Information Criteria (BIC). The results observed in this table, in general, corroborate the decision to use linear models; Table S2: Summary of the cross-validation analysis based on the application of two distinct methods recommended for use in time series, where the sequential phenomenon observed in the data must be respected [97,98]. The methods used here were fixed-window and rolling-origin (or expanding-window). In summary, the following quantities are presented: root mean square of the residuals (RMSE) for the training and test bases, as well as the ratio between test and training as a comparison metric. Values around 1 demonstrate stability and generalization capacity of the model. Values greater than 1.5 are considered indicators of overestimation and values less than 0.5 can be considered indicators of underfitting of the model; Table S3: Summary of the multivariate analysis of variance applied to the PCoA Groups to evaluated the significance of the groups observed for each side of the South Atlantic Ocean (SAO); Figure S1: Time series of catches of tuna, sharks and billfish caught in the South East Atlantic Ocean between 1978 and 2018; Figure S2: Time series of catches of tuna, sharks and billfish caught in the South West Atlantic Ocean between 1978 and 2018; Figure S3: Exploratory analysis of the Mean Temperature of Catches and Sea Surface Temperatures variables for South East Atlantic Ocean; Figure S4: Exploratory analysis of the Mean Temperature of Catches and Sea Surface Temperatures variables for South West Atlantic Ocean; Figure S5: Diagnostics analysis of the model fitted to the comparison between both sides of the South Atlantic Ocean (SAO)—ANCOVA. Top-left panel—Histogram of the residuals, indicating an approximately symmetric distribution centered at zero. Top-right panel—Dispersion plot of the residuals as a function of the order of observations, with no evident patterns of autocorrelation or heteroscedasticity. Bottom panel—Quantile plot (QQ plot) with simulated 95% envelope (blue dashed lines), comparing the residuals with the theoretical normal distribution. The general adherence to the reference line suggests that the assumption of normality of the residuals is adequate; Figure S6: Diagnostics analysis of the model fitted to the Mean Temperature of the Catches (MTC) for South West Atlantic Ocean. Top-left panel—Histogram of the residuals, indicating an approximately symmetric distribution centered at zero. Top-right panel—Dispersion plot of the residuals as a function of the order of observations, with no evident patterns of autocorrelation or heteroscedasticity. Bottom panel—Quantile plot (QQ plot) with simulated 95% envelope (blue dashed lines), comparing the residuals with the theoretical normal distribution. The general adherence to the reference line suggests that the assumption of normality of the residuals is adequate; Figure S7: Diagnostics analysis of the model fitted to the Sea Surface Temperature (SST) for South West Atlantic Ocean. Top-left panel—Histogram of the residuals, indicating an approximately symmetric distribution centered at zero. Top-right panel—Dispersion plot of the residuals as a function of the order of observations, with no evident patterns of autocorrelation or heteroscedasticity. Bottom panel—Quantile plot (QQ plot) with simulated 95% envelope (blue dashed lines), comparing the residuals with the theoretical normal distribution. The general adherence to the reference line suggests that the assumption of normality of the residuals is adequate; Figure S8: Diagnostics analysis of the model fitted to the Annual Transport Volume of the Brazil Current (BCt) for South West Atlantic Ocean. Top-left panel—Histogram of the residuals, indicating an approximately symmetric distribution centered at zero. Top-right panel—Dispersion plot of the residuals as a function of the order of observations, with no evident patterns of autocorrelation or heteroscedasticity. Bottom panel—Quantile plot (QQ plot) with simulated 95% envelope (blue dashed lines), comparing the residuals with the theoretical normal distribution. The general adherence to the reference line suggests that the assumption of normality of the residuals is adequate; Figure S9: Diagnostics analysis of the model fitted to the Mean Temperature of the Catches (MTC) for South East Atlantic Ocean. Top-left panel—Histogram of the residuals, indicating an approximately symmetric distribution centered at zero. Top-right panel—Dispersion plot of the residuals as a function of the order of observations, with no evident patterns of autocorrelation or heteroscedasticity. Bottom panel—Quantile plot (QQ plot) with simulated 95% envelope (blue dashed lines), comparing the residuals with the theoretical normal distribution. The general adherence to the reference line suggests that the assumption of normality of the residuals is adequate; Figure S10: Diagnostics analysis of the model fitted to the Sea Surface Temperature (SST) for South East Atlantic Ocean. Top-left panel—Histogram of the residuals, indicating an approximately symmetric distribution centered at zero. Top-right panel—Dispersion plot of the residuals as a function of the order of observations, with no evident patterns of autocorrelation or heteroscedasticity. Bottom panel—Quantile plot (QQ plot) with simulated 95% envelope (blue dashed lines), comparing the residuals with the theoretical normal distribution. The general adherence to the reference line suggests that the assumption of normality of the residuals is adequate; Figure S11: Diagnostics analysis of the models fitted to the Mean Temperature of the Catches (MTC) for South East Atlantic Ocean. Left-panels: Linear model. Right-panels: Non-Linear model. Top panels—Quantile plot (QQ plot) with simulated 95% envelope (blue dashed lines), comparing the residuals with the theoretical normal distribution. The general adherence to the reference line suggests that the assumption of normality of the residuals is adequate. Middle-panels—Histogram of the residuals, indicating an approximately symmetric distribution centered at zero. Bottom-panels—Dispersion plot of the residuals as a function of the order of observations, with no evident patterns of autocorrelation or heteroscedasticity; Figure S12: Diagnostics analysis of the models fitted to the Sea Surface Temperature (SST) for South East Atlantic Ocean. Left-panels: Linear model. Right-panels: Non-Linear model. Top panels—Quantile plot (QQ plot) with simulated 95% envelope (blue dashed lines), comparing the residuals with the theoretical normal distribution. The general adherence to the reference line suggests that the assumption of normality of the residuals is adequate. Middle-panels—Histogram of the residuals, indicating an approximately symmetric distribution centered at zero. Bottom-panels—Dispersion plot of the residuals as a function of the order of observations, with no evident patterns of autocorrelation or heteroscedasticity; Figure S13: Diagnostics analysis of the models fitted to the Mean Temperature of the Catches (MTC) for South West Atlantic Ocean. Left-panels: Linear model. Right-panels: Non-Linear model. Top panels—Quantile plot (QQ plot) with simulated 95% envelope (blue dashed lines), comparing the residuals with the theoretical normal distribution. The general adherence to the reference line suggests that the assumption of normality of the residuals is adequate. Middle-panels—Histogram of the residuals, indicating an approximately symmetric distribution centered at zero. Bottom-panels—Dispersion plot of the residuals as a function of the order of observations, with no evident patterns of autocorrelation or heteroscedasticity; Figure S14: Diagnostics analysis of the models fitted to the Sea Surface Temperature (SST) for South West Atlantic Ocean. Left-panels: Linear model. Right-panels: Non-Linear model. Top panels—Quantile plot (QQ plot) with simulated 95% envelope (blue dashed lines), comparing the residuals with the theoretical normal distribution. The general adherence to the reference line suggests that the assumption of normality of the residuals is adequate. Middle-panels—Histogram of the residuals, indicating an approximately symmetric distribution centered at zero. Bottom-panels—Dispersion plot of the residuals as a function of the order of observations, with no evident patterns of autocorrelation or heteroscedasticity; Figure S15: Diagnostics analysis of the models fitted to the Annual Transport Volume of the Brazil Current (BCt) for South West Atlantic Ocean. Left-panels: Linear model. Right-panels: Non-Linear model. Top panels—Quantile plot (QQ plot) with simulated 95% envelope (blue dashed lines), comparing the residuals with the theoretical normal distribution. The general adherence to the reference line suggests that the assumption of normality of the residuals is adequate. Middle-panels—Histogram of the residuals, indicating an approximately symmetric distribution centered at zero. Bottom-panels—Dispersion plot of the residuals as a function of the order of observations, with no evident patterns of autocorrelation or heteroscedasticity; Figure S16: Annual contribution to total beta-diversity (YCBD) computed between 1978 and 2018 for both sides of the South Atlantic Ocean; Figure S17: Biomass gains and losses in the catches of the pelagic species caught in the both sides of the South Atlantic Ocean; Figure S18: Summary of the discriminant analysis applied as a post-hoc test of the multivariate analysis of variance to evaluate the differences among the PCoA Groups for the South West Atlantic Ocean; Figure S19: Summary of the discriminant analysis applied as a post-hoc test of the multivariate analysis of variance to evaluate the differences among the PCoA Groups for the South East Atlantic Ocean.
