# Supplementary material for: Increasing Sea Surface Temperatures Driving Widespread Tropicalization in South Atlantic Pelagic Fisheries
Source: Biology (Basel). 2025 Aug 13;14(8):1039. doi: 10.3390/biology14081039 (PMC12383501; doi:10.3390/biology14081039)
Supplement: Supplementary file 1 [file biology-14-01039-s001.zip › biology-3696440-supplementary.pdf]

Increasing Sea Surface Temperatures Driving Widespread Tropicalization in South Atlantic Pelagic Fisheries

Rodrigo Sant'Ana [1,*], Daniel Thá [1], Lea-Anne Henry [2], Rafael Schroeder [1,3] and José Angel Alvarez Perez [1]

[1] Laboratório de Estudos Marinhos Aplicados, Escola Politécnica, Universidade do Vale do Itajaí (UNIVALI), Rua Uruguai 458, Itajaí 88302-901, Brazil; daniel.tha@kralingen.com.br (D.T.); schroederichthys@gmail.com (R.S.); angel.perez@univali.br (J.A.A.P.)

[2] School of GeoSciences, University of Edinburgh, King's Buildings, James Hutton Road, Edinburgh EH9 3FE, UK; l.henry@ed.ac.uk

[3] Centro Interdisciplinar de Investigação Marinha e Ambiental (CIIMAR), Terminal de Cruzeiros do Porto de Leixões, Avenida General Norton de Matos S/N, 4550-208 Matosinhos, Portugal

* Correspondence: rsantana@univali.br

Supplementary Information

This supplementary material summarizes a series of information and analyses consolidated within the scope of this manuscript. A total of three Tables and 19 Figures are presented here, which generally present diagnostics of the models implemented in this work, as well as some additional descriptive and inferential analyses.

To better understand this supplementary material, a general description is presented below, organized by analytical structure linked to the original document.

The descriptive/exploratory component of the data includes supplementary Figures S1, S2, S3, and S4. Figures S1 and S2 present the time series of tuna and tuna-like catches derived from the ICCAT database and consolidated for the analyses implemented in this study. Figures S3 and S4 summarize the individual behavior of the MTC and SST variables, following an approximately normal distribution, for both sides of the South Atlantic Ocean.

Supplementary Table S1 presents a summary of the analysis of the model structures (e.g., linear or nonlinear) incorporated into the analysis of this study and as described in the materials and methods of this manuscript. As observed in Table S1, the adjustment of generalized additive models with the proposed structure generally did not demonstrate improvements that would justify their use over the application of simpler models, such as linear regression models. With the exception of the SST response variable adjusted for the Southeast Atlantic Ocean, where the AIC and adjusted R-squared tended to show better results with the implementation of nonlinear models, all other models tended to apply the linear structure. However, when observing Figures S11 to S15, which summarize the analysis of the residuals of the fitted models, both with linear and nonlinear structures, no observable differences were observed in the behavior of the residuals, which, in both cases, assume a normal distribution, centered at zero, and constant variance.

Regarding the diagnostics of the implemented models, now fitted with a linear structure, a cross-validation analysis was implemented based on the implementation of two distinct methods recommended for use in time series [98,99]. The methods used were fixed-window and expanding-window. Supplementary Table S2 summarizes the results of both analyses conducted for the different models applied to both sides of the Atlantic Ocean. The observed ratio between the division of the RMSE obtained by the training and testing bases shows that the implemented models exhibit acceptable stability and generalization capacity, indicating neither overestimation nor underestimation. Additionally, for each of the fitted models, a residual analysis was performed to assess the quality of the fits and compliance with the assumptions of normality and independence. Supplementary Figures S5 to S10 summarize these results, demonstrating compliance with these assumptions, with normally distributed residuals around the mean 0, randomly distributed throughout the data series, and generally within the limits estimated by the simulated envelope analysis.
As a result of the principal coordinates analysis, the formation of clusters across the time series was also subjected to an inferential approach, applying a multivariate analysis of variance. As observed in

Supplementary Table S3, the clusters formed on both sides of the South Atlantic Ocean showed significant differences, which can be further demonstrated in the discriminant analysis implemented as a post-multivariate analysis of variance test (Supplementary Figures S18 and S19). Finally, as discussed in the manuscript itself, supplementary Figures S16 and S17 provide support for the discussions held within the original document.

Tables

Supplementary Table S1: Summary of the comparison between Linear and Non-Linear models applied to the Mean Temperature of the Catch (MTC), Sea Surface Temperature (SST) and Annual Transport Volume of the Brazil Current (BCt) time-series. South East Atlantic Ocean (SEAO) – MTC and SST were evaluated; South West Atlantic Ocean (SWAO) – MTC, SST and BCt were evaluated. For each method were presented the adjusted r-squared (Adj. r^2), Akaike Information Criteria (AIC) and Bayesian Information Criteria (BIC). The results observed in this table, in general, corroborate the decision to use linear models.

Ocean side	Variable	Method	Adj. r^2	AIC	BIC
SEAO	MTC	Linear model	0.1087	-46.808	-41.667
		Non-Linear model	0.1087	-46.808	-41.667
	SST	Linear model	0.3012	-39.219	-34.219
		Non-Linear model	0.3928	-43.168	-34.382
SWAO	MTC	Linear model	0.3422	-14.053	-8.913
		Non-Linear model	0.3422	-14.053	-8.913
	SST	Linear model	0.4336	-63.138	-57.998
		Non-Linear model	0.4336	-63.138	-57.998
	BCt	Linear model	0.4255	130.570	134.227
		Non-Linear model	0.4255	130.570	134.227

Supplementary Table S2: Summary of the cross-validation analysis based on the application of two distinct methods recommended for use in time series, where the sequential phenomenon observed in the data must be respected [98,99]. The methods used here were fixed-window and rolling-origin (or expanding-window). In summary, the following quantities are presented: root mean square of the residuals (RMSE) for the training and test bases, as well as the ratio between test and training as a comparison metric. Values around 1 demonstrate stability and generalization capacity of the model. Values greater than 1.5 are considered indicators of overestimation and values less than 0.5 can be considered indicators of underfitting of the model.

Ocean side	Variable	Method	RMSE train	RMSE test	Ratio
SEAO	MTC	Fixed-window	0.127	0.155	1.22
		Expanding-window	0.127	0.149	1.17
	SST	Fixed-window	0.139	0.167	1.20
		Expanding-window	0.139	0.133	0.95
SWAO	MTC	Fixed-window	0.189	0.194	1.02
		Expanding-window	0,189	0.186	0.98
	SST	Fixed-window	0.104	0.125	1.20
		Expanding-window	0.104	0.114	1.09
	BCt	Fixed-window	2.920	3.140	1.07
		Expanding-window	2.920	2.860	0.98

Supplementary Table S3: Summary of the multivariate analysis of variance applied to the PCoA Groups to evaluated the significance of the groups observed for each side of the South Atlantic Ocean (SAO).

Ocean side	Statistic	D.F.	Test Statistic	Approx. F	Num. D.F.	Den. D.F.	p-value
SEAO	Pillai	2	1.8089	6.995	46	34	2.968E-08
	Wilks	2	0.0057	8.519	46	32	4.918E-09
	Hotelling-Lawley	2	31.5352	10.283	46	30	1.138E-09
	Roy	2	26.0486	19.253	23	17	4.042E-08
SWAO	Pillai	4	3.6001	5.409	100	60	3.703E-11
	Wilks	4	0.00002	7.735	100	50	6.732E-13
	Hotelling-Lawley	4	108.8428	11.429	100	42	2.489E-14
	Roy	4	75.0832	45.049	25	15	4.303E-10

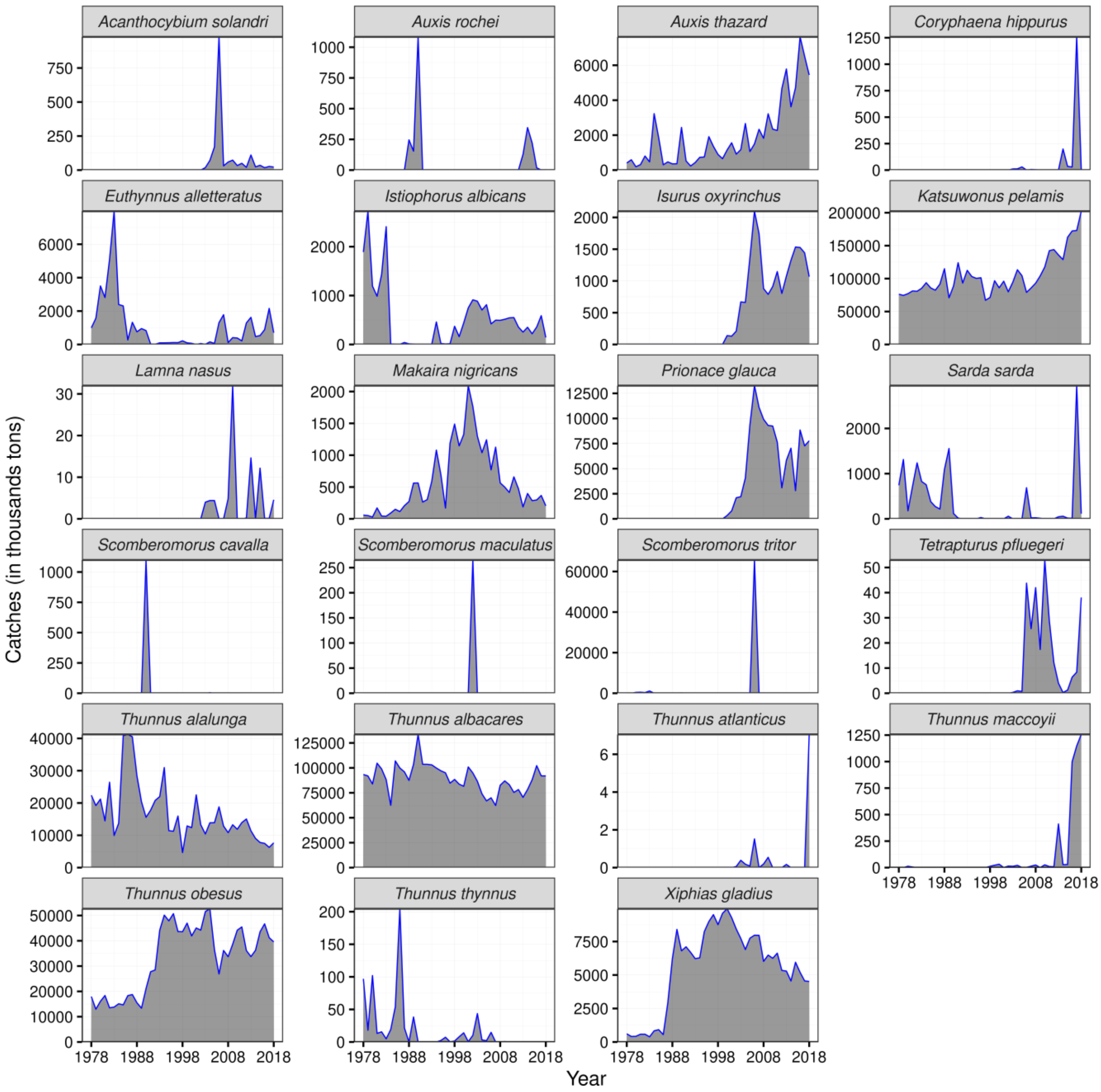

Supplementary Figure S1: Time series of catches of tuna, sharks and billfish caught in the South East Atlantic Ocean between 1978 and 2018.

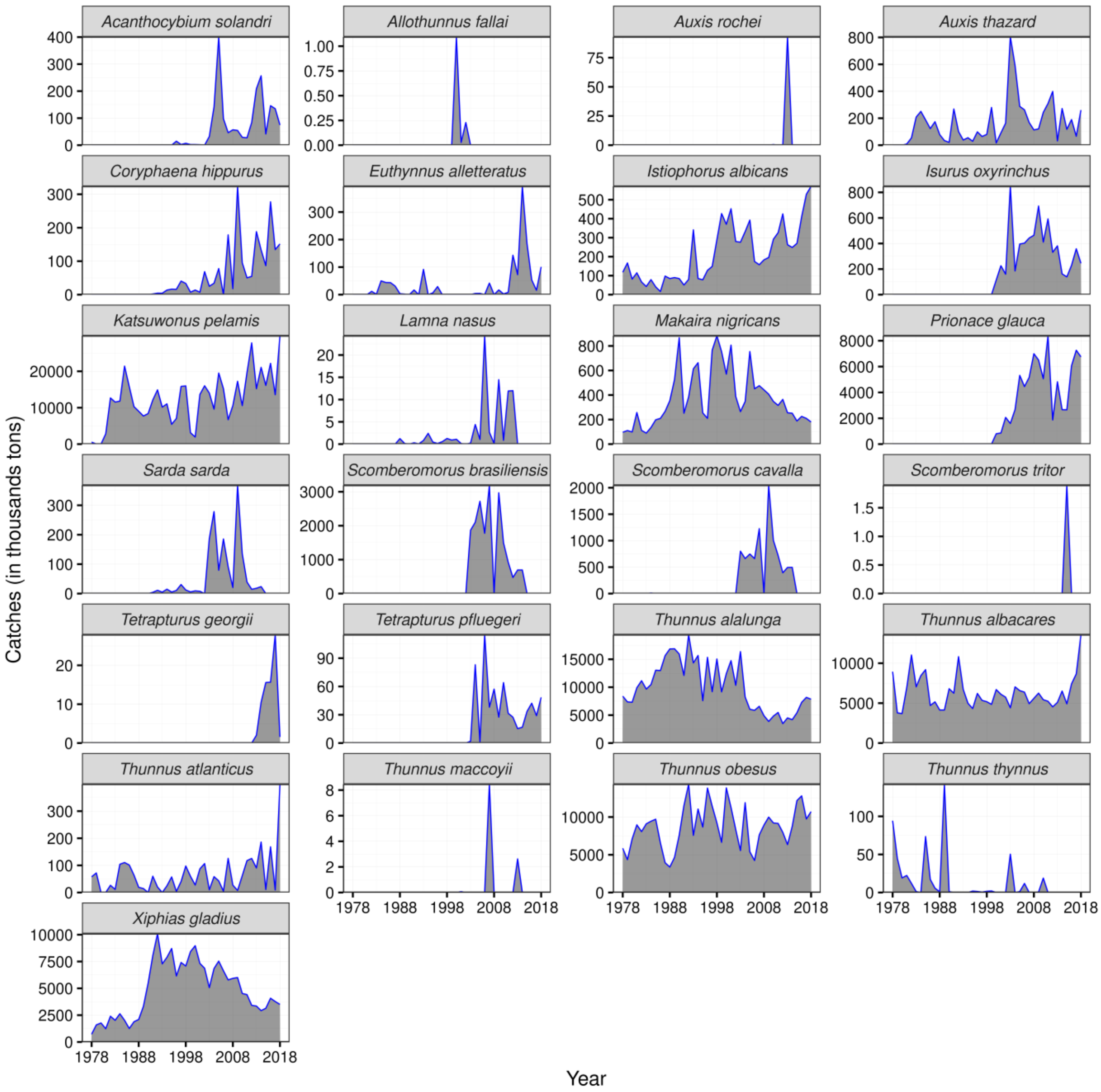

Supplementary Figure S2: Time series of catches of tuna, sharks and billfish caught in the South West Atlantic Ocean between 1978 and 2018.

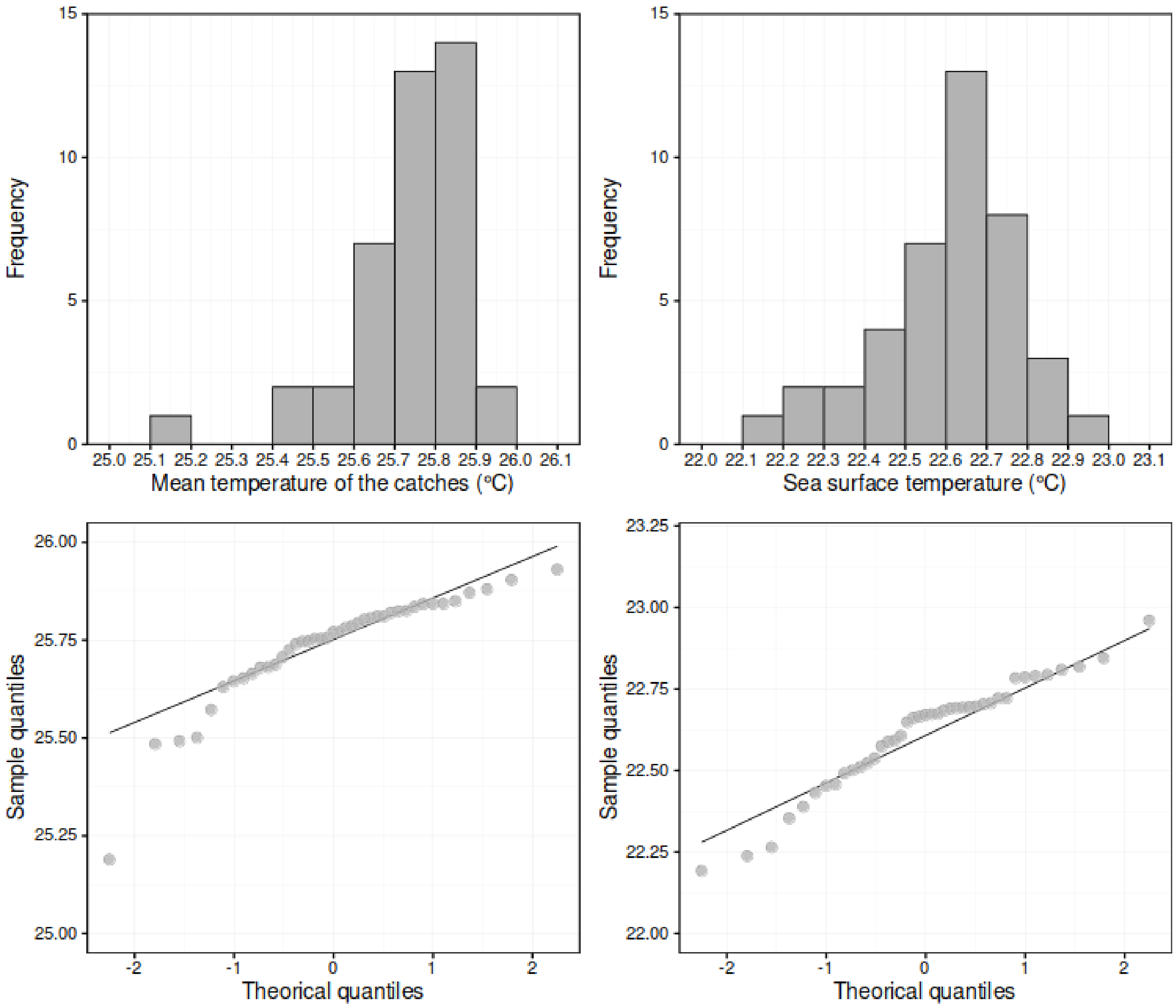

Supplementary Figure S3: Exploratory analysis of the Mean Temperature of Catches and Sea Surface Temperatures variables for South East Atlantic Ocean.

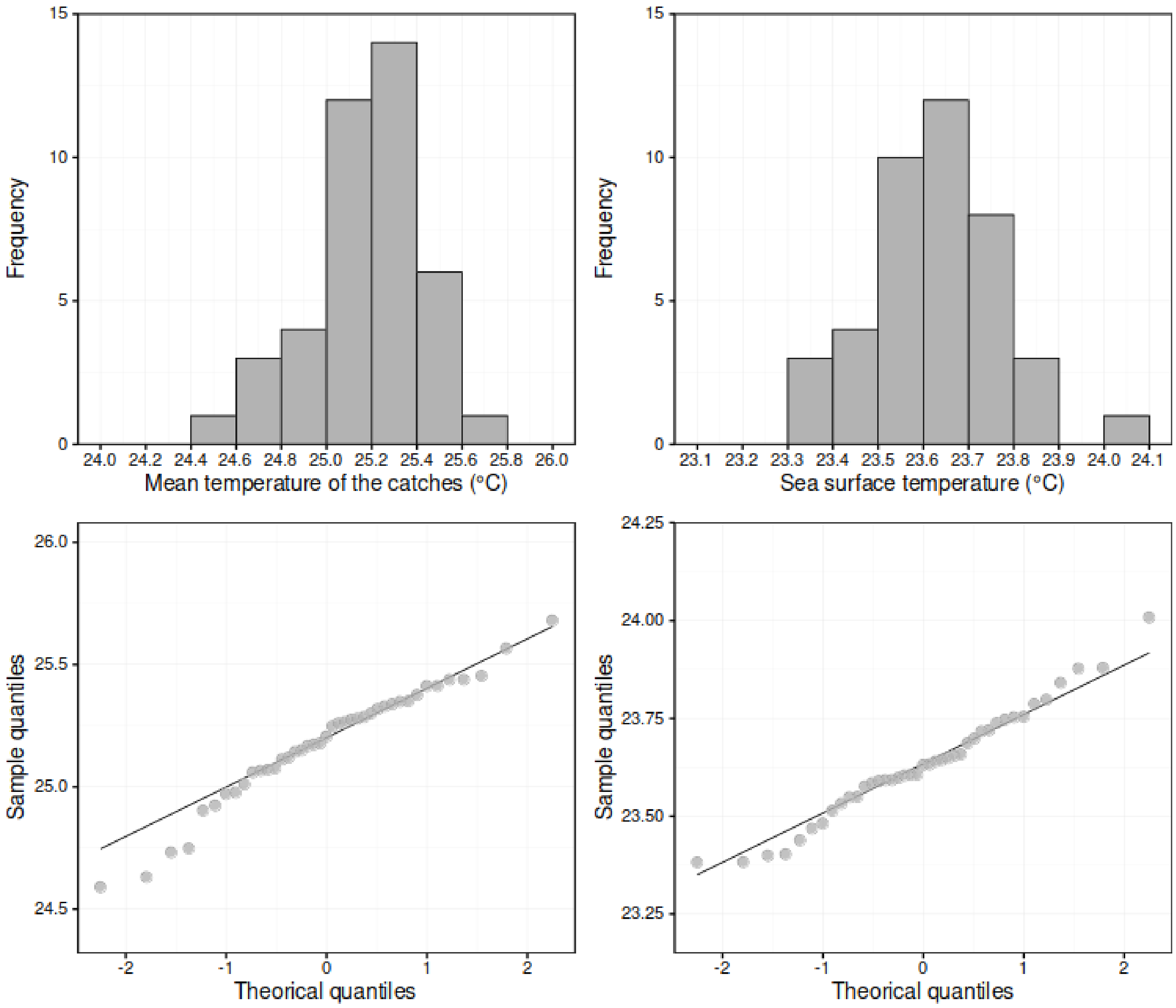

Supplementary Figure S4: Exploratory analysis of the Mean Temperature of Catches and Sea Surface Temperatures variables for South West Atlantic Ocean.

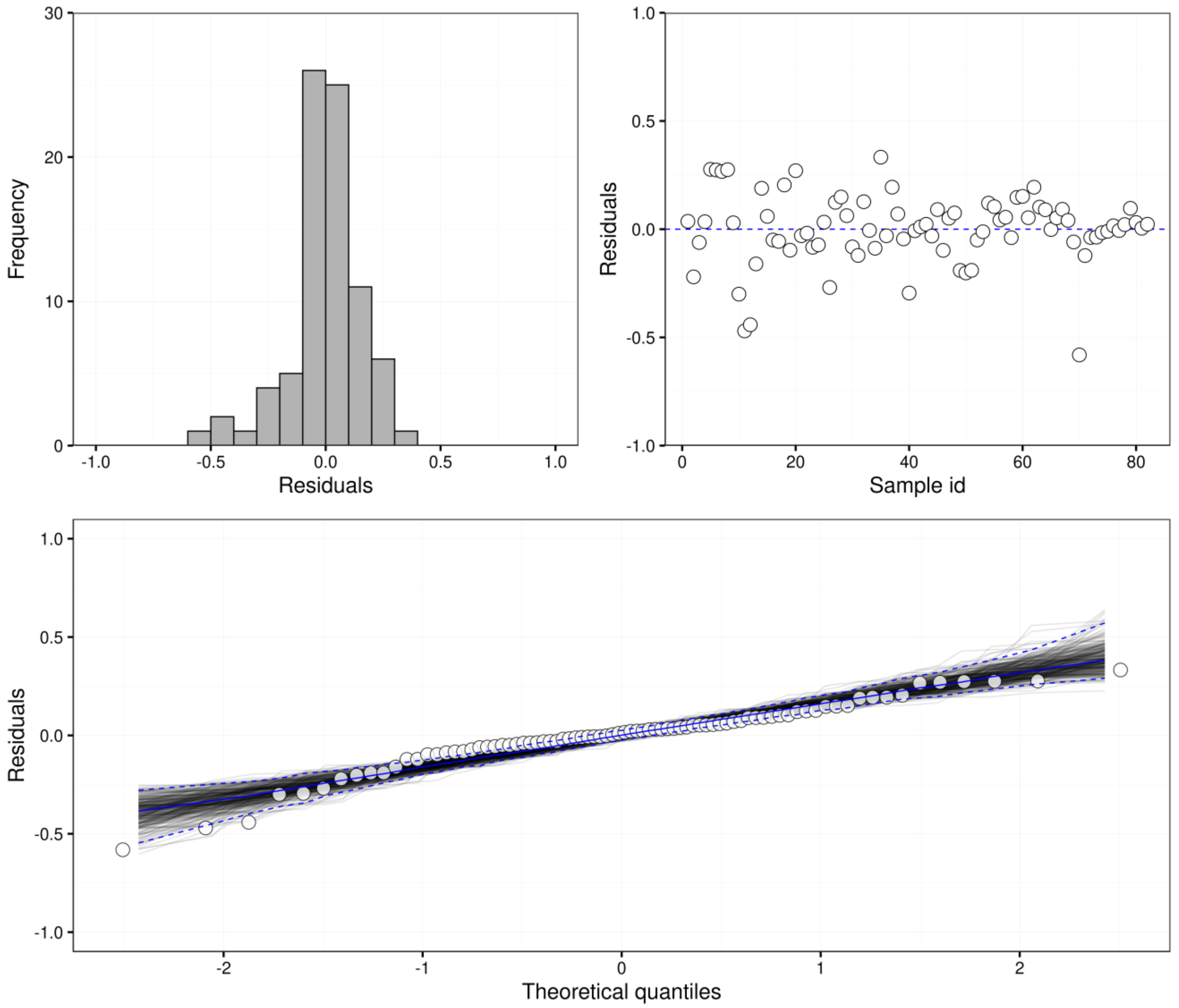

Supplementary Figure S5: Diagnostics analysis of the model fitted to the comparison between both sides of the South Atlantic Ocean (SAO) - ANCOVA. Top-left panel – Histogram of the residuals, indicating an approximately symmetric distribution centered at zero. Top-right panel – Dispersion plot of the residuals as a function of the order of observations, with no evident patterns of autocorrelation or heteroscedasticity. Bottom panel - Quantile plot (QQ plot) with simulated 95% envelope (blue dashed lines), comparing the residuals with the theoretical normal distribution. The general adherence to the reference line suggests that the assumption of normality of the residuals is adequate.

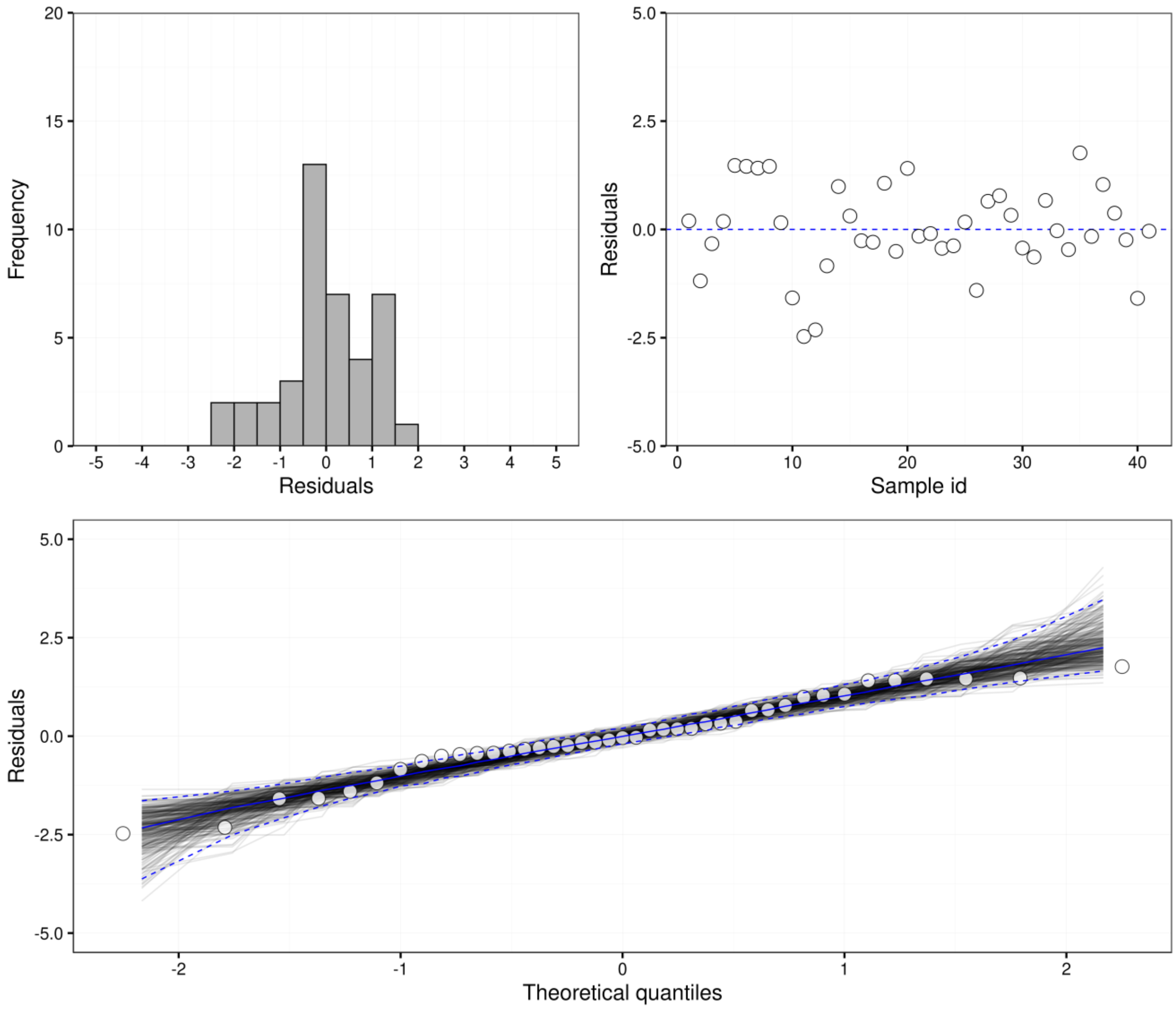

Supplementary Figure S6: Diagnostics analysis of the model fitted to the Mean Temperature of the Catches (MTC) for South West Atlantic Ocean. Top-left panel – Histogram of the residuals, indicating an approximately symmetric distribution centered at zero. Top-right panel – Dispersion plot of the residuals as a function of the order of observations, with no evident patterns of autocorrelation or heteroscedasticity. Bottom panel - Quantile plot (QQ plot) with simulated 95% envelope (blue dashed lines), comparing the residuals with the theoretical normal distribution. The general adherence to the reference line suggests that the assumption of normality of the residuals is adequate.

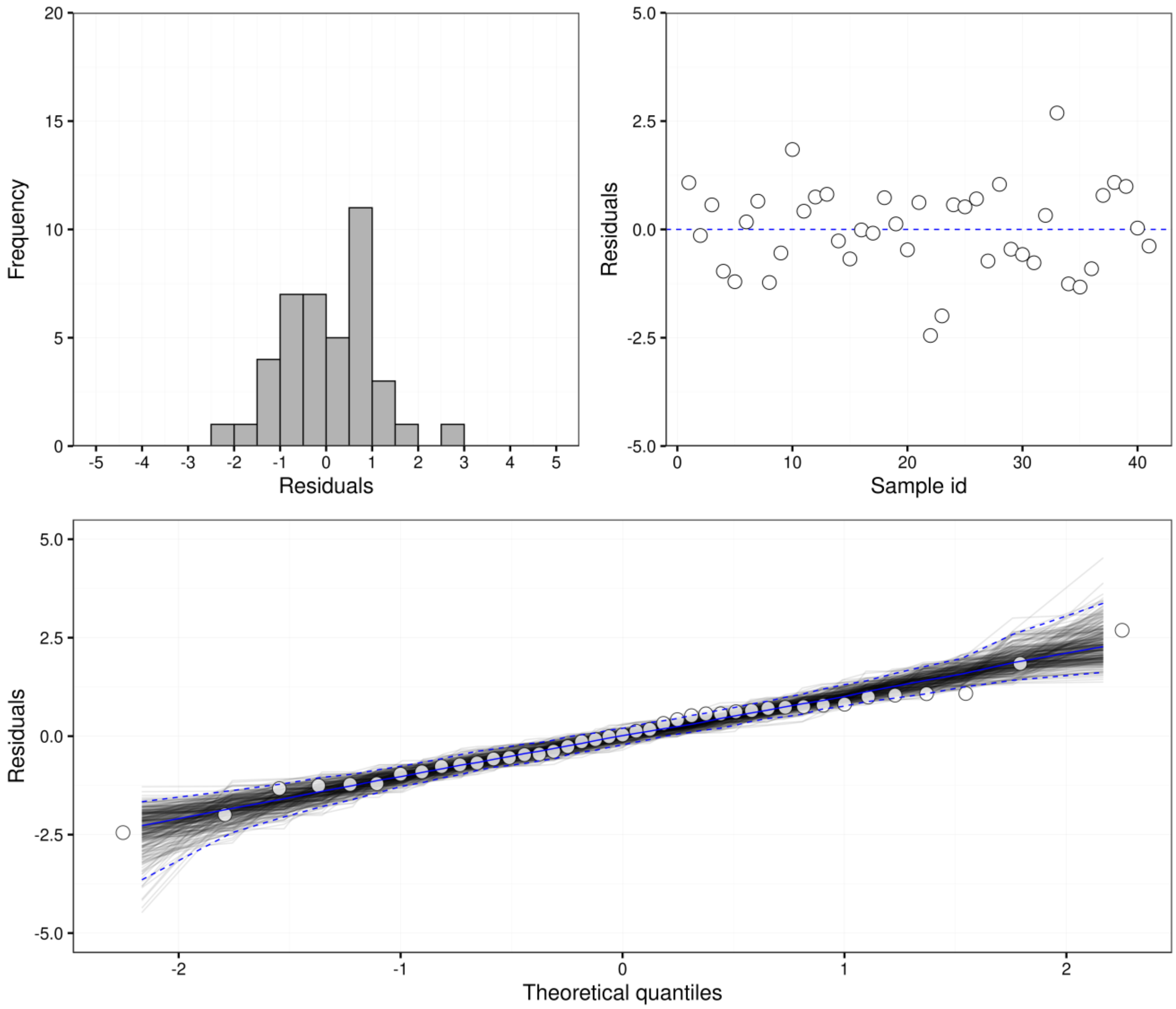

Supplementary Figure S7: Diagnostics analysis of the model fitted to the Sea Surface Temperature (SST) for South West Atlantic Ocean. Top-left panel – Histogram of the residuals, indicating an approximately symmetric distribution centered at zero. Top-right panel – Dispersion plot of the residuals as a function of the order of observations, with no evident patterns of autocorrelation or heteroscedasticity. Bottom panel - Quantile plot (QQ plot) with simulated 95% envelope (blue dashed lines), comparing the residuals with the theoretical normal distribution. The general adherence to the reference line suggests that the assumption of normality of the residuals is adequate.

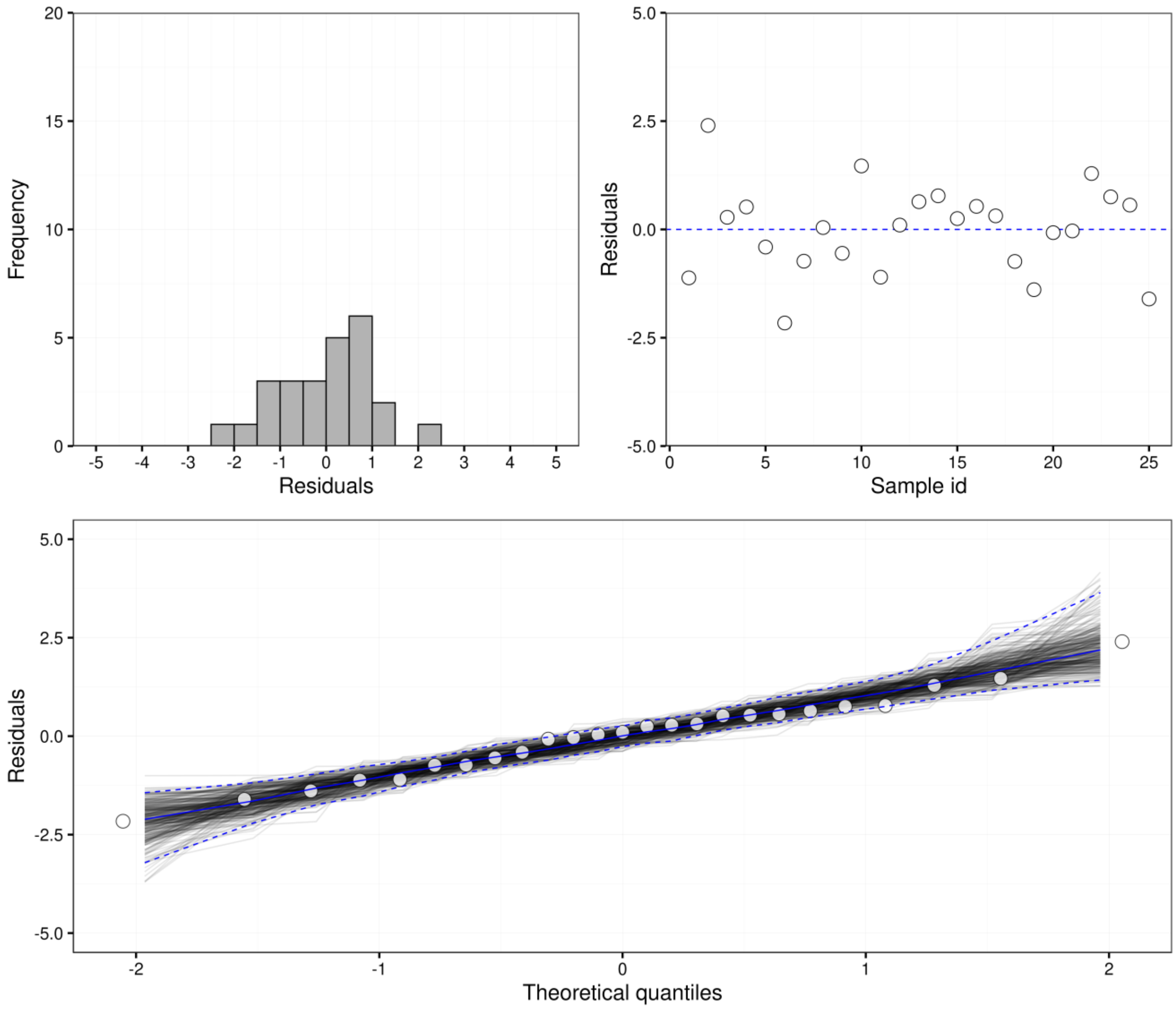

Supplementary Figure S8: Diagnostics analysis of the model fitted to the Annual Transport Volume of the Brazil Current (BCt) for South West Atlantic Ocean. Top-left panel – Histogram of the residuals, indicating an approximately symmetric distribution centered at zero. Top-right panel – Dispersion plot of the residuals as a function of the order of observations, with no evident patterns of autocorrelation or heteroscedasticity. Bottom panel - Quantile plot (QQ plot) with simulated 95% envelope (blue dashed lines), comparing the residuals with the theoretical normal distribution. The general adherence to the reference line suggests that the assumption of normality of the residuals is adequate.

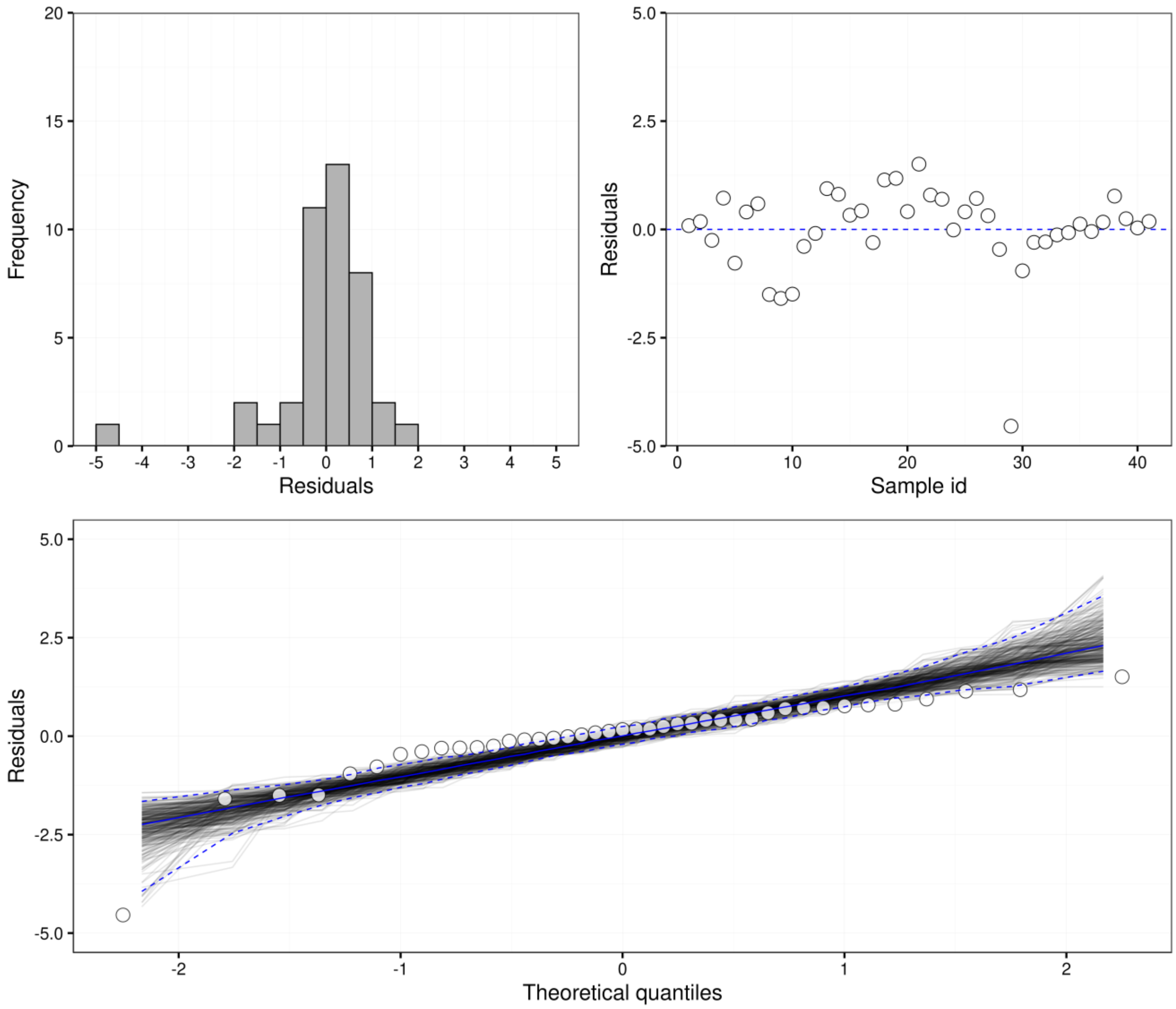

Supplementary Figure S9: Diagnostics analysis of the model fitted to the Mean Temperature of the Catches (MTC) for South East Atlantic Ocean. Top-left panel – Histogram of the residuals, indicating an approximately symmetric distribution centered at zero. Top-right panel – Dispersion plot of the residuals as a function of the order of observations, with no evident patterns of autocorrelation or heteroscedasticity. Bottom panel - Quantile plot (QQ plot) with simulated 95% envelope (blue dashed lines), comparing the residuals with the theoretical normal distribution. The general adherence to the reference line suggests that the assumption of normality of the residuals is adequate.

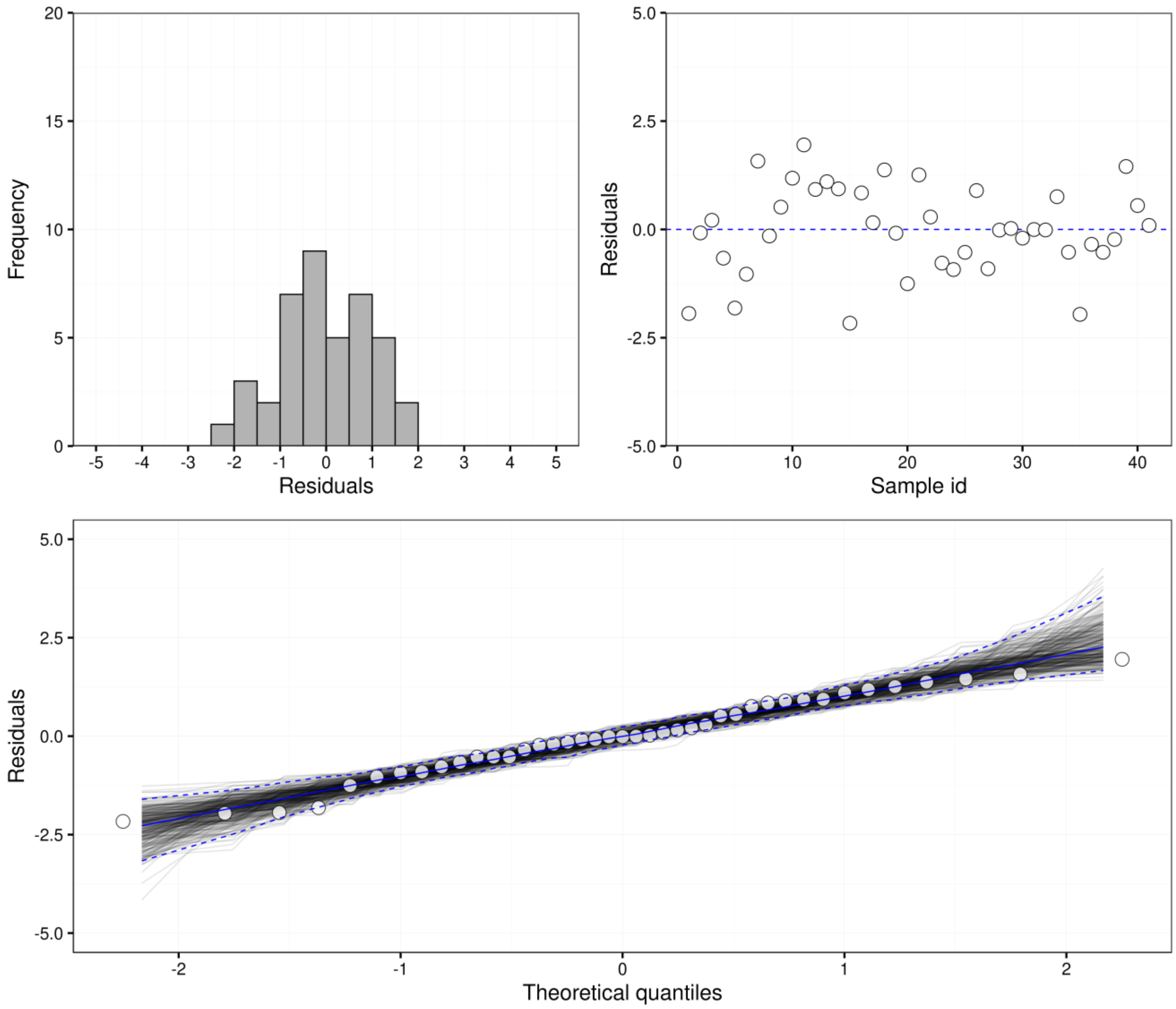

Supplementary Figure S10: Diagnostics analysis of the model fitted to the Sea Surface Temperature (SST) for South East Atlantic Ocean. Top-left panel – Histogram of the residuals, indicating an approximately symmetric distribution centered at zero. Top-right panel – Dispersion plot of the residuals as a function of the order of observations, with no evident patterns of autocorrelation or heteroscedasticity. Bottom panel - Quantile plot (QQ plot) with simulated 95% envelope (blue dashed lines), comparing the residuals with the theoretical normal distribution. The general adherence to the reference line suggests that the assumption of normality of the residuals is adequate.

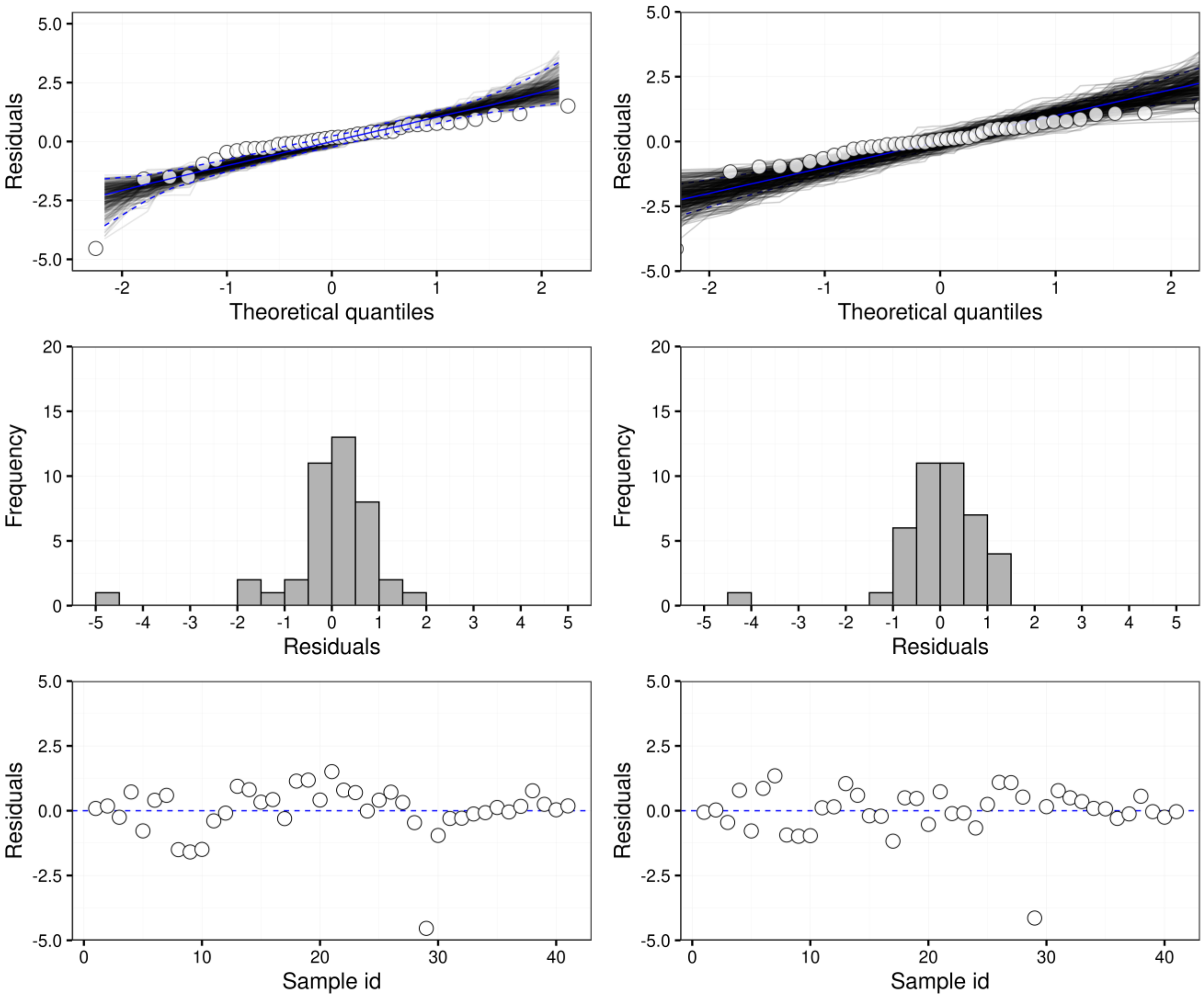

Supplementary Figure S11: Diagnostics analysis of the models fitted to the Mean Temperature of the Catches (MTC) for South East Atlantic Ocean. Left-panels: Linear model. Right-panels: Non-Linear model. Top panels – Quantile plot (QQ plot) with simulated 95% envelope (blue dashed lines), comparing the residuals with the theoretical normal distribution. The general adherence to the reference line suggests that the assumption of normality of the residuals is adequate. Middle-panels - Histogram of the residuals, indicating an approximately symmetric distribution centered at zero. Bottom-panels – Dispersion plot of the residuals as a function of the order of observations, with no evident patterns of autocorrelation or heteroscedasticity.

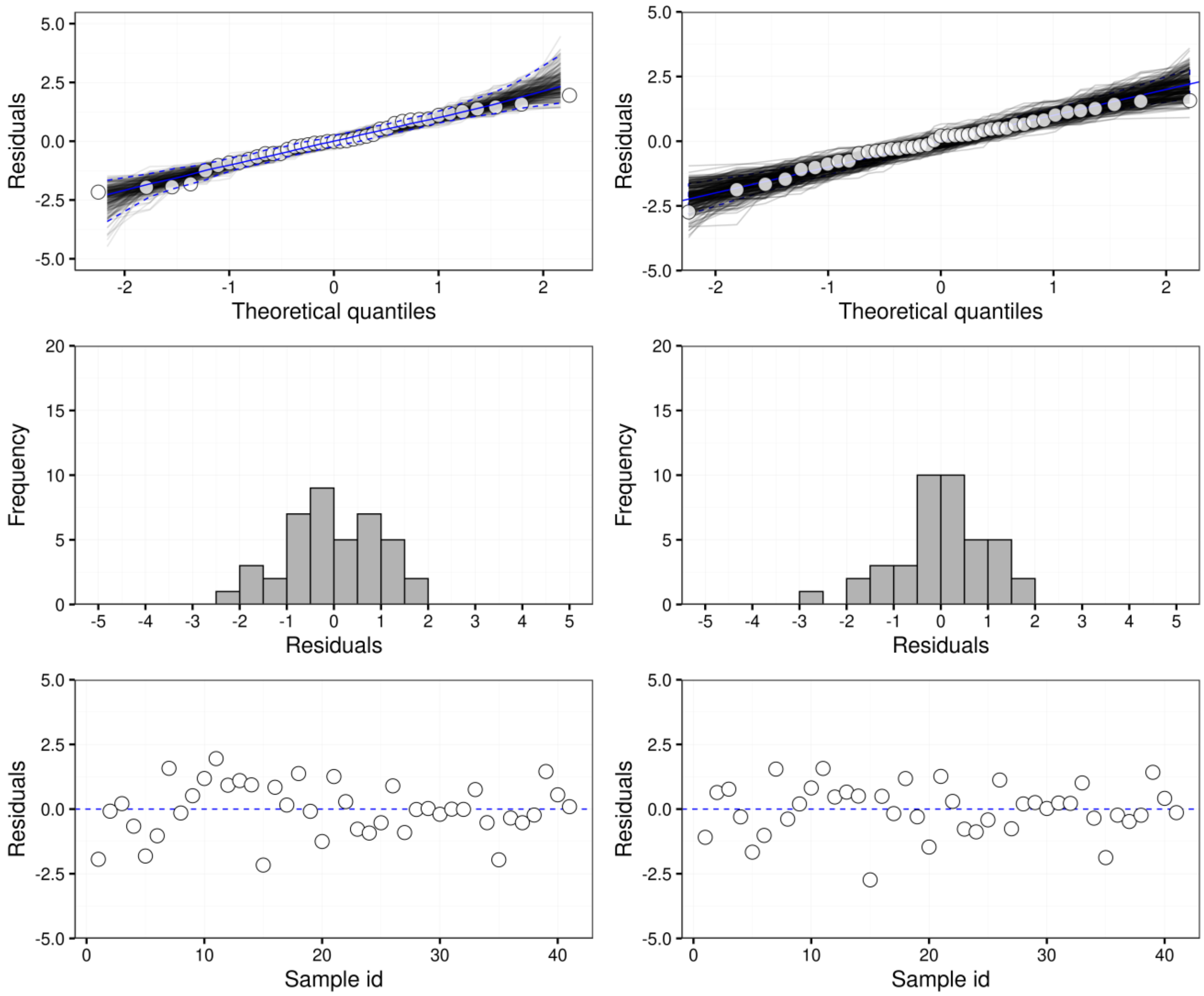

Supplementary Figure S12: Diagnostics analysis of the models fitted to the Sea Surface Temperature (SST) for South East Atlantic Ocean. Left-panels: Linear model. Right-panels: Non-Linear model. Top panels – Quantile plot (QQ plot) with simulated 95% envelope (blue dashed lines), comparing the residuals with the theoretical normal distribution. The general adherence to the reference line suggests that the assumption of normality of the residuals is adequate. Middle-panels - Histogram of the residuals, indicating an approximately symmetric distribution centered at zero. Bottom-panels – Dispersion plot of the residuals as a function of the order of observations, with no evident patterns of autocorrelation or heteroscedasticity.

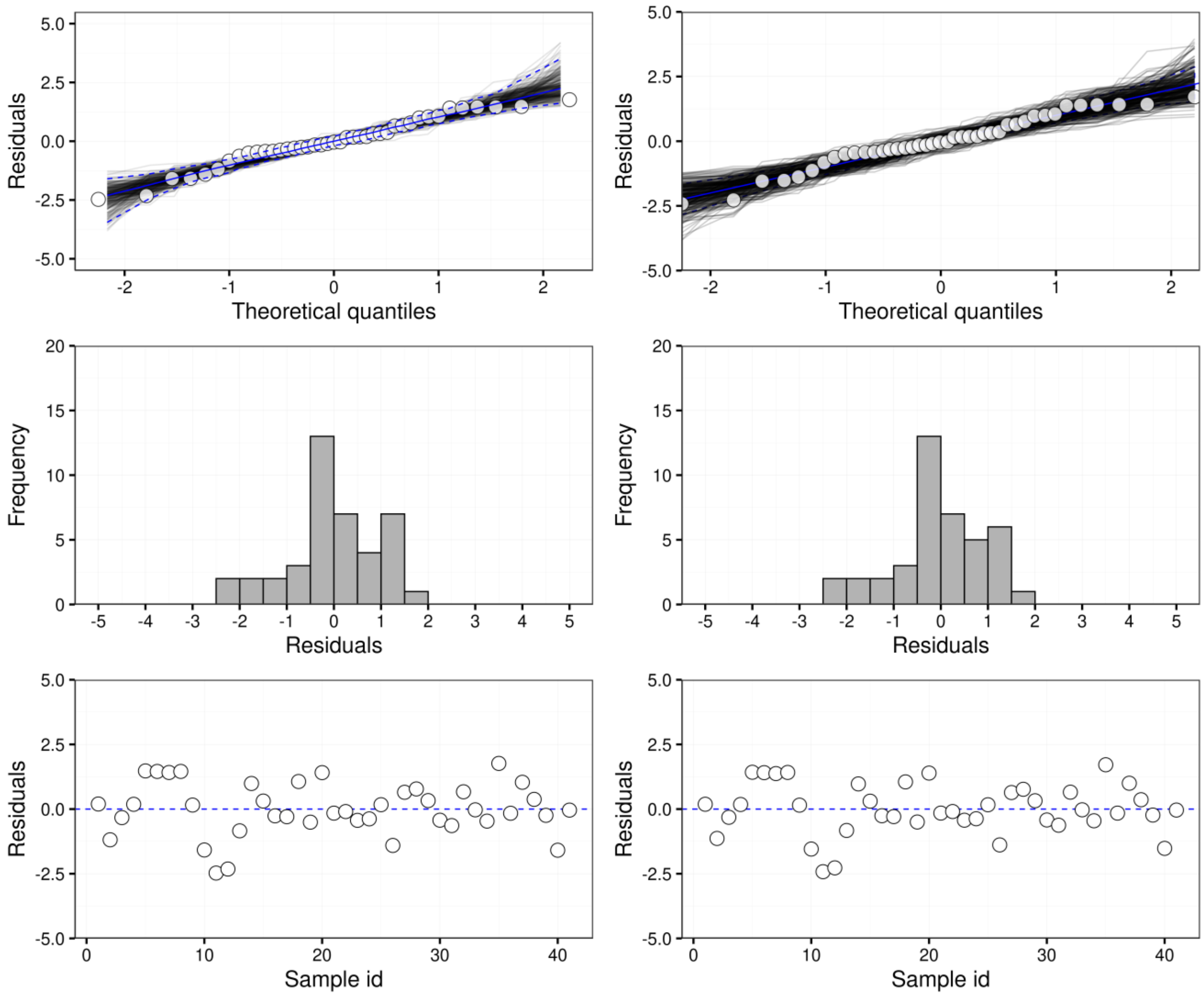

Supplementary Figure S13: Diagnostics analysis of the models fitted to the Mean Temperature of the Catches (MTC) for South West Atlantic Ocean. Left-panels: Linear model. Right-panels: Non-Linear model. Top panels – Quantile plot (QQ plot) with simulated 95% envelope (blue dashed lines), comparing the residuals with the theoretical normal distribution. The general adherence to the reference line suggests that the assumption of normality of the residuals is adequate. Middle-panels - Histogram of the residuals, indicating an approximately symmetric distribution centered at zero. Bottom-panels – Dispersion plot of the residuals as a function of the order of observations, with no evident patterns of autocorrelation or heteroscedasticity.

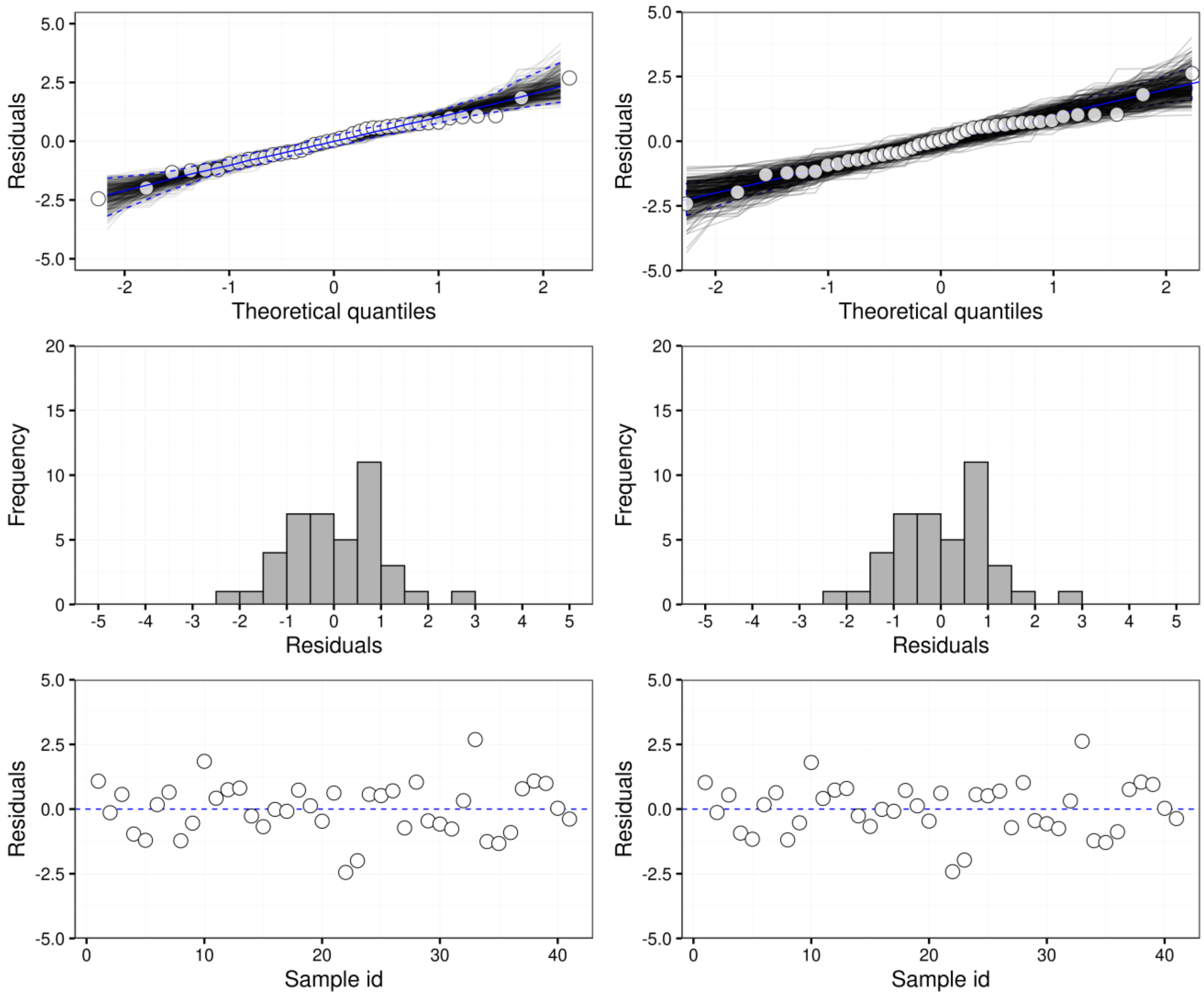

Supplementary Figure S14: Diagnostics analysis of the models fitted to the Sea Surface Temperature (SST) for South West Atlantic Ocean. Left-panels: Linear model. Right-panels: Non-Linear model. Top panels – Quantile plot (QQ plot) with simulated 95% envelope (blue dashed lines), comparing the residuals with the theoretical normal distribution. The general adherence to the reference line suggests that the assumption of normality of the residuals is adequate. Middle-panels - Histogram of the residuals, indicating an approximately symmetric distribution centered at zero. Bottom-panels – Dispersion plot of the residuals as a function of the order of observations, with no evident patterns of autocorrelation or heteroscedasticity.

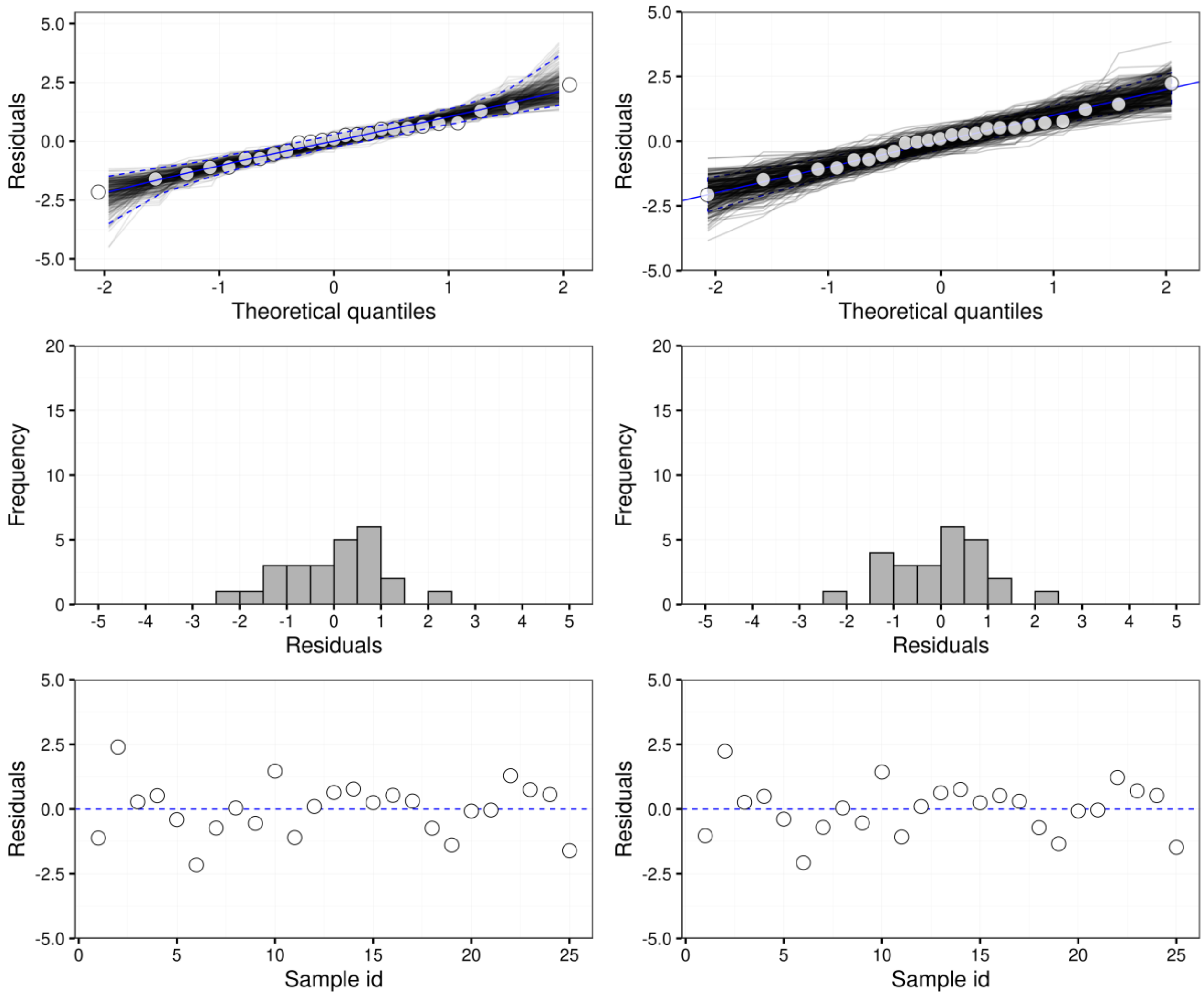

Supplementary Figure S15: Diagnostics analysis of the models fitted to the Annual Transport Volume of the Brazil Current (BCt) for South West Atlantic Ocean. Left-panels: Linear model. Right-panels: Non-Linear model. Top panels – Quantile plot (QQ plot) with simulated 95% envelope (blue dashed lines), comparing the residuals with the theoretical normal distribution. The general adherence to the reference line suggests that the assumption of normality of the residuals is adequate. Middle-panels - Histogram of the residuals, indicating an approximately symmetric distribution centered at zero. Bottom-panels – Dispersion plot of the residuals as a function of the order of observations, with no evident patterns of autocorrelation or heteroscedasticity.

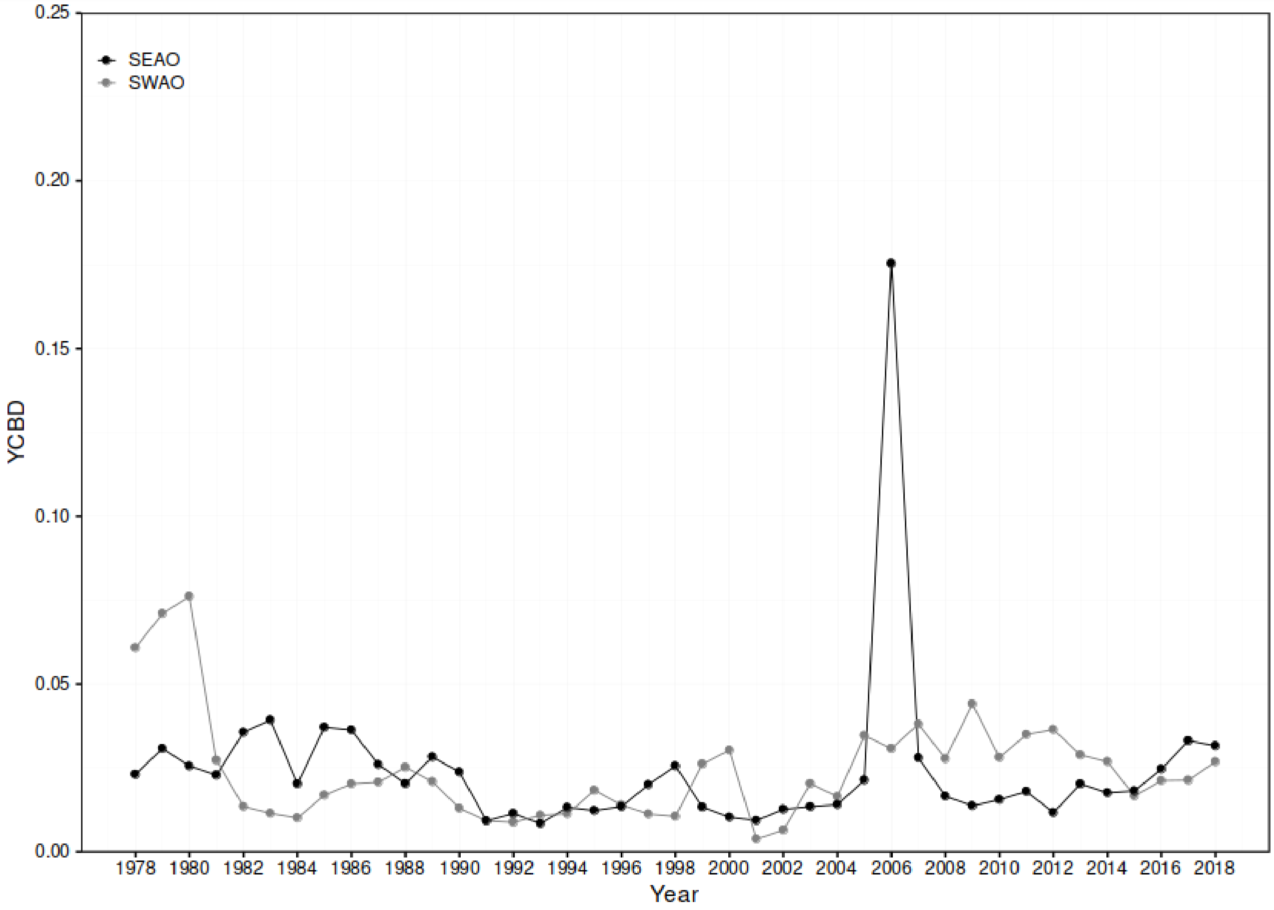

Supplementary Figure S16: Annual contribution to total beta-diversity (YCBD) computed between 1978 and 2018 for both sides of the South Atlantic Ocean.

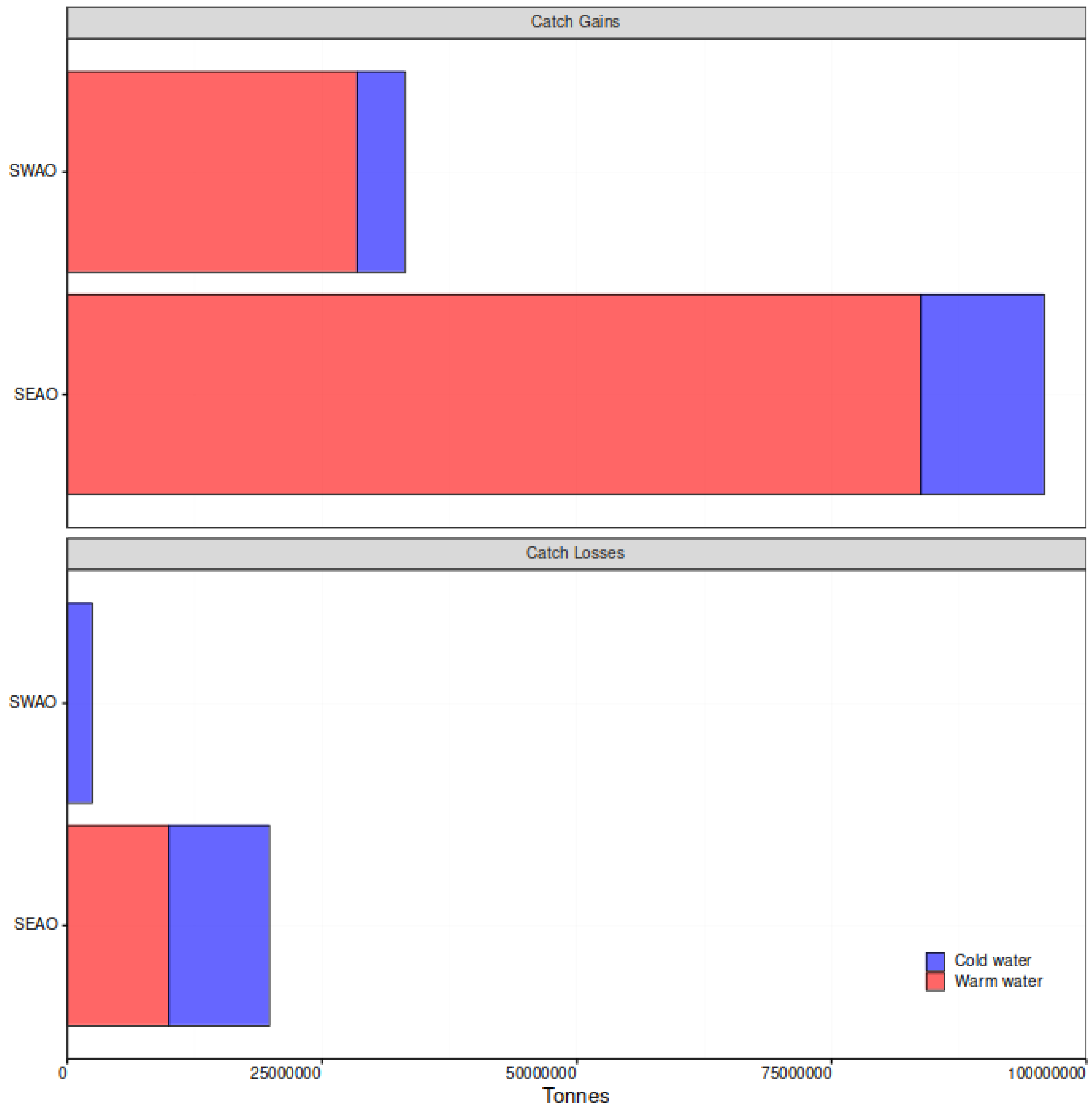

Supplementary Figure S17: Biomass gains and losses in the catches of the pelagic species caught in the both sides of the South Atlantic Ocean.

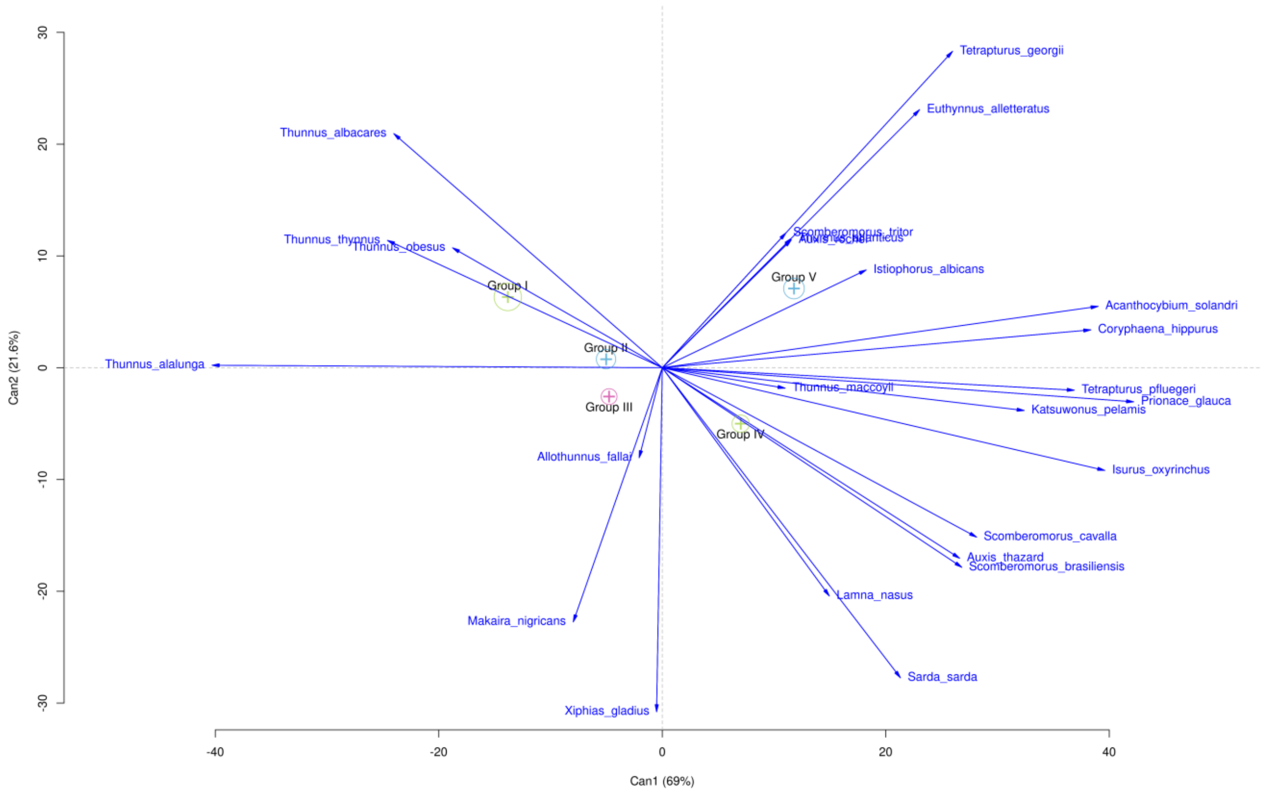

Supplementary Figure S18: Summary of the discriminant analysis applied as a post-hoc test of the multivariate analysis of variance to evaluate the differences among the PCoA Groups for the South West Atlantic Ocean.

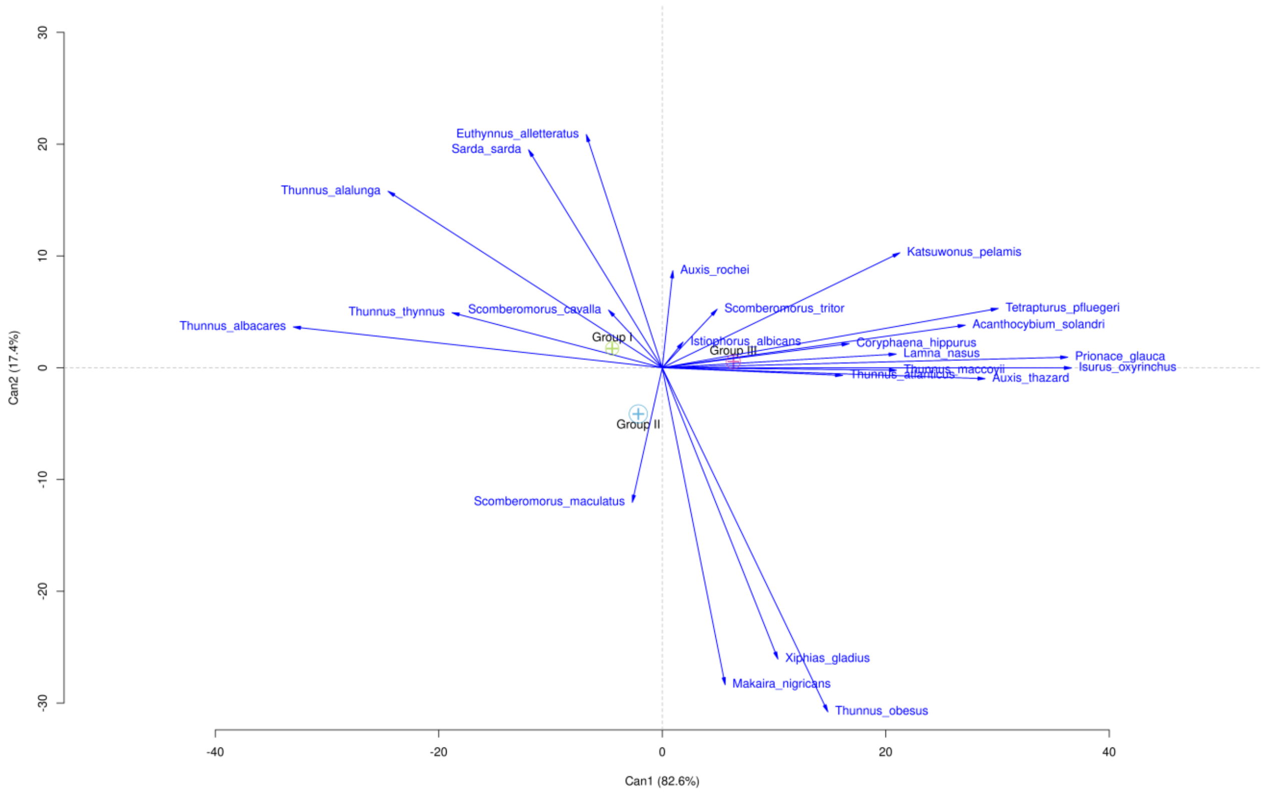

Supplementary Figure S19: Summary of the discriminant analysis applied as a post-hoc test of the multivariate analysis of variance to evaluate the differences among the PCoA Groups for the South East Atlantic Ocean.
